# Investigating the interrelationship of vitamin K and vitamin D status on bone density: results from the VITamin D and OmegA-3 TriaL

**DOI:** 10.1093/jbmrpl/ziag110

**Published:** 2026-07-09

**Authors:** Sharon H Chou, Sarah L Booth, Nancy R Cook, Eunjung Kim, Gregory Kotler, Xueyan Fu, Julie E Buring, JoAnn E Manson, Meryl S LeBoff

**Affiliations:** Division of Endocrinology, Diabetes and Hypertension, Department of Medicine, Brigham and Women’s Hospital, Boston, MA 02115, United States; Harvard Medical School, Boston, MA 02115, United States; Jean Mayer USDA Human Nutrition Research Center on Aging, Tufts University, Boston, MA 02111, United States; Harvard Medical School, Boston, MA 02115, United States; Division of Preventive Medicine, Department of Medicine, Brigham and Women’s Hospital, Harvard Medical School, Boston, MA 02215, United States; Department of Epidemiology, Harvard T.H. Chan School of Public Health, Boston, MA 02115, United States; Harvard Medical School, Boston, MA 02115, United States; Division of Preventive Medicine, Department of Medicine, Brigham and Women’s Hospital, Harvard Medical School, Boston, MA 02215, United States; Division of Preventive Medicine, Department of Medicine, Brigham and Women’s Hospital, Harvard Medical School, Boston, MA 02215, United States; Jean Mayer USDA Human Nutrition Research Center on Aging, Tufts University, Boston, MA 02111, United States; Harvard Medical School, Boston, MA 02115, United States; Division of Preventive Medicine, Department of Medicine, Brigham and Women’s Hospital, Harvard Medical School, Boston, MA 02215, United States; Department of Epidemiology, Harvard T.H. Chan School of Public Health, Boston, MA 02115, United States; Harvard Medical School, Boston, MA 02115, United States; Division of Preventive Medicine, Department of Medicine, Brigham and Women’s Hospital, Harvard Medical School, Boston, MA 02215, United States; Department of Epidemiology, Harvard T.H. Chan School of Public Health, Boston, MA 02115, United States; Division of Endocrinology, Diabetes and Hypertension, Department of Medicine, Brigham and Women’s Hospital, Boston, MA 02115, United States; Harvard Medical School, Boston, MA 02115, United States

**Keywords:** vitamin D, vitamin K, bone density, bone architecture, bone turnover

## Abstract

Sufficient intake of both vitamin D and K may be necessary for bone health. Although the synthesis of osteocalcin (OC) is stimulated by vitamin D, OC needs to be carboxylated, or activated, by vitamin K to deposit calcium into bone. In the VITamin D and OmegA-3 TriaL (VITAL), there were no differences in 2-yr changes in areal BMD (aBMD) at the spine, hip, or whole body in 771 U.S. participants (687 with follow-up data) randomized to supplemental vitamin D_3_ (2000 IU/d) vs placebo. In this ancillary study, we investigated whether baseline vitamin K status was associated with changes in bone density and structure and whether baseline vitamin K status modified the effects of supplemental vitamin D on aBMD changes. Higher baseline vitamin K status, as assessed by blood levels of phylloquinone, percent undercarboxylated OC, and dephosphorylated undercarboxylated matrix Gla protein, was not associated with favorable 2-yr changes in aBMD at the spine, hip, or whole body as assessed by DXA, trabecular bone score, or volumetric BMD (vBMD) and bone architecture outcomes at the radius and tibia as assessed by peripheral QCT. The effects of supplemental vitamin D on aBMD were not modified by baseline measures of vitamin K. In VITAL, higher vitamin K status, even when accompanied by supplemental vitamin D, did not prevent bone loss.

Clinical Trial Registration No.: NCT04573946 (ClinicalTrials.gov).

## Introduction

Vitamin K may play a necessary role in the effects of vitamin D on bone metabolism. Although vitamin D stimulates calcium absorption by the intestines and production of osteocalcin (OC) by osteoblasts, vitamin K is needed to carboxylate and activate OC. Carboxylated OC then directs calcium deposition to the bone.^1^

We have previously reported in an ancillary study to the VITamin D and OmegA-3 TriaL (VITAL) that supplemental vitamin D_3_ (2000 IU/d) vs placebo for 2 yr did not benefit areal BMD (aBMD) at the spine, hip, or whole body in 771 U.S. men aged ≥50 yr and women aged ≥55 yr.^2^ In the overall cohort of 25 871 U.S. participants, we also found that vitamin D supplementation did not reduce incidence of fractures over a median of 5.3 yr of follow-up.^3^ Because approximately 60% of older U.S. men and 40% of women have inadequate vitamin K intake,^4^ we assessed whether low vitamin K status may explain the lack of response to vitamin D supplementation.

Observational studies have linked low vitamin K intake or low serum levels with low bone density^5–8^ and increased fracture risk.^9–12^ Most clinical trials investigating effects of supplemental vitamin K1, or phylloquinone (PK), found no effects on aBMD and included low doses of supplemental vitamin D (≤400 IU/d).^13–16^ Currently, there are limited data on the interaction of high doses of supplemental vitamin D and vitamin K status on measures of bone density, structure, and architecture. In particular, high doses of supplemental vitamin D may create an imbalance between these micronutrients.

While 25OHD is considered the standard vitamin D measurement, there is no standard biomarker for vitamin K. Phylloquinone, the main form of vitamin K found in green vegetables and plant oils in Western diets, can be measured in the serum. However, menaquinones (MK, or vitamin K2), mostly produced from PK by intestinal bacteria, are usually not detectable in blood unless supplemented.^17^ While PK levels provide a measure of dietary intake and absorption, other biomarkers, such as undercarboxylated OC (ucOC) and dephosphorylated undercarboxylated matrix Gla protein ([dp]ucMGP), reflect vitamin K activity. As vitamin K is required to carboxylate OC and MGP, high levels of ucOC and (dp)ucMGP signify low vitamin K status.^17^

For this ancillary study titled “VITAL: Interrelationship of Vitamin D and Vitamin K on Bone” (NCT04573946), we investigated the relationship between baseline vitamin K status, alone or in combination with vitamin D status, and 2-yr changes in bone density, structure, and architecture. In the VITAL bone health sub-cohort of 771 participants (687 participants with follow-up data), we hypothesized that low baseline vitamin K status, alone or in combination with low baseline vitamin D status, would be associated with increased bone loss and deterioration of bone structure and architecture over 2 yr. We also hypothesized that baseline low vitamin K status would attenuate the benefits of supplemental vitamin D on 2-yr changes in bone density.

## Materials and methods

### Trial design

The parent VITAL trial was a 2 × 2 factorial, double-blinded, randomized, placebo-controlled trial that investigated the effects of supplemental vitamin D_3_ and/or marine omega-3 fatty acid, compared to placebo, on the primary prevention of cancer and cardiovascular disease in 25 871 U.S. men aged ≥50 yr and women aged ≥55 yr.^18^^,^^19^ Over a median treatment duration of 5.3 yr, neither supplemental vitamin D nor marine omega-3 fatty acid reduced incidence of cancer or cardiovascular disease in the overall cohort, although there were certain subgroups who benefited. This trial protocol has been detailed previously.^18–20^ Exclusion criteria for the overall cohort included history of cancer (except nonmelanoma skin cancer) and cardiovascular disease, given the aims of the parent trial, as well as hypercalcemia, parathyroid disorder, renal failure, severe liver disease, or other serious illness for safety reasons.

VITAL had a hybrid design. In addition to the overall cohort of 25 871 participants, a sub-cohort of 1054 participants were recruited for in-person clinical assessments. The only inclusion criteria were living within driving distance of Boston, Massachusetts, and being able to provide informed consent. Within this sub-cohort, participants were recruited for additional bone health measures, if they were not on bisphosphonates within the past 2 yr or denosumab, teriparatide, raloxifene, tamoxifen, or systemic estrogens within the past year. Participants on vitamin K supplements, multivitamins, or medications that influence vitamin K metabolism were not excluded from this ancillary study. This ancillary study was conducted in 771 participants who had in-person evaluations of bone health and fasting morning blood collections at baseline and 2-yr follow-up.

### Ancillary study endpoints

The primary outcomes of this ancillary study were 2-yr changes in aBMD, as assessed by DXA. In the bone health sub-cohort, 771 participants underwent DXA (Discovery W, APEX Software Version 4.2, Hologic) at baseline between November 2012 and March 2014, and 687 participants completed scans at 2-yr follow-up between October 2014 and July 2016 (89% retention). Nineteen participants were started on pharmacologic treatment for osteoporosis and were not eligible for follow-up, and 65 participants did not complete 2-yr follow-up visits. Endpoints included 2-yr changes in aBMD at the LS (L1-L4), nondominant femur (TH and FN), and whole body. Precision at our site is excellent with the following least significant changes: 0.024 g/cm^2^ at the spine, 0.017 g/cm^2^ at the TH, 0.021 g/cm^2^ at the FN, and 0.008 g/cm^2^ at whole body for men and 0.010 g/cm^2^ at the whole body for women.^2^

Secondary outcomes included 2-yr changes in bone structure and architecture, as assessed by trabecular bone score (TBS) and peripheral QCT (pQCT) scan. DXA images of the spine were used to generate TBS using TBS iNsight software (version 2.1; Medimaps Group). Trabecular bone score uses a quantitative algorithm to measure gray-level variation of the LS DXA image to estimate 3D textural characteristics, providing an assessment of bone microarchitecture. Trabecular bone score has been found to predict fracture risk independent of aBMD.^21^^,^^22^ As per manufacturer’s guidelines, at baseline, TBS was not performed in participants with BMI ≥37 kg/m^2^ (54 participants) or ≤15 kg/m^2^ (no participants).

Within the bone health sub-cohort, 667 participants underwent pQCT scan (XCT 3000; Stratec Medizintechnik GmbH) at baseline and 600 participants at 2-yr follow-up (89% retention) to measure bone structure and architecture at the nondominant radius and tibia. These endpoints included total, trabecular, and cortical vBMD, cortical thickness, and bone strength measures (bone strength index [BSI] and polar stress strength index [SSI]). Total and trabecular vBMD and BSI were measured at the 4% radius and tibia sites, while cortical vBMD, cortical thickness, and SSI were measured at the 33% radius and 38% tibia sites. For pQCT measures, our percent coefficient of variation (CV) was very good, ranging from 0.02% to 2.87% at the radius and tibia. Our procedural protocols for both DXA and pQCT have been previously detailed.^2^

We also explored whether baseline vitamin K status was associated with 2-yr changes in bone turnover markers (BTMs), including amino-terminal propeptide of type 1 collagen (P1NP), total OC, and C-telopeptide (CTX).

### Measurement of blood samples

Fasting blood samples were obtained at baseline and 2-yr follow-up and batched to measure the following vitamin K biomarkers: PK (*n* = 770), ucOC (*n* = 753), total OC (*n* = 753), and (dp)ucMGP (*n* = 771). Reverse-phased HPLC with post-column, chemical reduction of PK followed by fluorometric detection was used to analyze PK levels. The CV for PK was 7.9%. Sandwich ELISAs (IDS-iSYS Multi-Discipline Automated System) were used to measure levels of ucOC, total OC, and (dp)ucMGP. The CV for ucOC and total OC were 7.6%-8.8%, and the CV for (dp)ucMGP was 9.9%. %ucOC was calculated from ucOC and total OC, and high levels of %ucOC indicate decreased vitamin K activity. Dephosphorylated undercarboxylated matrix Gla protein is a measure of the inactive form of the protein. Vitamin K biomarkers were analyzed at the Vitamin K Laboratory at Jean Mayer USDA Human Nutrition Research Center on Aging at Tufts University, a participant in the vitamin K external quality assurance scheme.^23^

25OHD levels were also measured in the blood samples at baseline and 2-yr follow-up, matched by season. To minimize potential batch effects, all baseline and year 2 sample pairs of 25OHD were measured at the same time in tandem by liquid chromatography-tandem mass spectrometry by Quest Diagnostics. VITAL partook in the Vitamin D Standardization Program of the Centers for Disease Control and Prevention.^24^

Finally, fasting serum BTMs were also assessed at baseline and 2-yr follow-up. CTX levels were measured by enzyme immunoassay from Immunodiagnostic Systems Inc., and P1NP levels were determined by radioimmunoassay from Orion Diagnostica.

### Statistical analysis

We first examined the distribution of bone measures and applied normalizing and/or other transformations as needed. Repeated analysis with proc mixed model was used to estimate the association of baseline vitamin K status with changes in bone measures over time. We examined this using medians and clinical categories for biomarkers of exposure, and adjusted for age, sex, and randomization group. Although there are no established threshold for vitamin K sufficiency based on PK levels or undercarboxylated vitamin K-dependent proteins, we selected an arbitrary PK cut off of <0.5 nmol/L to indicate low levels.^17^^,^^25^ For (dp)ucMGP, we used the undetectable levels of (dp)ucMGP (≤300 pmol/L) to indicate high vitamin K status. We also examined effect modification of the randomized vitamin D intervention by baseline vitamin K status.

Based on observations in VITAL, which included 687 participants with data on 2-yr changes in aBMD,^2^ we had estimated 80% power to detect minimum differences of 0.72% change in FN aBMD, 0.71% change in spine aBMD, and 0.59% change in TH BMD for the observed effect of baseline vitamin K biomarker, divided at the median. We also estimated 80% power to detect minimum differences of 1.45% for FN aBMD, 1.42% for spine aBMD, and 1.18% for TH aBMD for interaction of the treatment effect with baseline vitamin K biomarkers above and below the median.

Statistical analyses were performed with SAS (SAS Institute). *p*-values <.05 were considered statistically significant. However, *p* values were not adjusted for multiple hypotheses testing; thus, all analyses, especially those that are exploratory, should be interpreted with caution.

## Results

### Baseline characteristics of the bone health sub-cohort

The baseline characteristics of the 771 participants in this bone health sub-cohort have been previously published.^2^ The mean (±SD) age of participants was 63.8 ± 6.1 yr, and the mean BMI was 27.2 ± 4.8 kg/m^2^. Exactly 46.7% of this population was female, and 83.4% were non-Hispanic White, reflecting demographics of the New England region. About 388 participants were randomized to active vitamin D_3_ intervention, and 383 were randomized to vitamin D placebo. Baseline characteristics were balanced between the 2 groups. Although personal vitamin D supplementation use was similar between the 2 groups, 25OHD levels were slightly higher in the placebo group at baseline.

The median PK level at baseline was 0.90 nmol/L. More participants in the age group of 65-74 yr old had higher PK levels (Table 1). There were no differences in distribution of sex, race, or BMI between participants with PK levels above or below the median. Participants with lower and higher PK levels at baseline had similar rates of personal history of fragility fracture, parental history of hip fracture, and history of fall in the past year. While there were more current tobacco users among the participants with lower PK levels, there were more daily alcohol users among the participants with higher PK levels. There was no difference in use of personal vitamin D supplements or calcium supplements in participants with baseline PK levels below or above the median.

**Table 1:** Baseline characteristics by phylloquinone (PK) levels.

Characteristics	PK <median (0.90 nmol/L)	PK ≥median (0.90 nmol/L)	*p*-value
**Gender**			.85
**Male**	186 (53.8%)	225 (53.1%)	
**Female**	160 (46.2%)	199 (46.9%)	
**Age**			.015
**50**-**54**	31 (9.0%)	22 (5.2%)	
**55**-**64**	195 (56.4%)	218 (51.4%)	
**65**-**74**	101 (29.2%)	165 (38.9%)	
**≥75**	19 (5.5%)	19 (4.5%)	
**Race**			.75
**Non**-**Hispanic White**	281 (83.1%)	348 (83.7%)	
**Black**	32 (9.5%)	35 (8.4%)	
**Hispanic**	13 (3.8%	13 (3.1%)	
**Other**	12 (3.6%)	20 (4.8%)	
**Body mass index, kg/m** ^**2**^			.58
**<18.5**	3 (0.9%)	4 (0.9%)	
**18.4**-**24.9**	99 (28.6%)	142 (33.5%)	
**25**-**29.9**	163 (47.1%)	176 (41.5%)	
**30**-**34.9**	53 (15.3%)	68 (16.0%)	
**≥35**	28 (8.1%)	34 (8.0%)	
**Tobacco use**			.037
**Never**	165 (48.1%)	218 (51.7%)	
**Past**	148 (43.1%)	186 (44.1%)	
**Current**	30 (8.7%)	18 (4.3%)	
**Alcohol use**			<.001
**Never**	79 (24.3%)	65 (16.2%)	
**<1 serving/wk**	21 (6.5%)	28 (7.0%)	
**1**-**6 servings/wk**	143 (44.0%)	152 (37.8%)	
**Daily**	82 (25.2%)	157 (39.1%)	
**History of fragility fracture, *n* (%)**	23 (6.6%)	38 (9.0%)	.24
**Parental history of hip fracture, *n* (%)**	43 (12.4%)	59 (13.9%)	.55
**History of fall in the last year, *n* (%)**	66 (20.2%)	75 (18.5%)	.55
**Multivitamin use, *n* (%)**	128 (39.8%)	187 (46.2%)	.083
**Calcium supplement use, *n* (%)**	53 (15.3%)	79 (18.6%)	.23
**Personal vitamin D supplement use, *n* (%)**	143 (41.3%)	183 (43.2%)	.61
**Completion of 2**-**yr follow, *n* (%)**	316 (91.3%)	392 (92.5%)	.57

Table 2 shows the distribution of vitamin K biomarkers in the bone health sub-cohort by treatment group. 91 participants (23.5%) in the vitamin D group and 77 participants (20.2%) in the vitamin D placebo group had very low PK levels of <0.5 nmol/L. Eight participants (2.1%) in the vitamin D group and 16 participants (4.2%) in the vitamin D placebo group had undetectable (dp)ucMGP levels (≤300 pmol/L), indicating robust vitamin K activity.

**Table 2:** Distribution of vitamin K biomarkers at baseline.

Biomarker	*N*	All (median [Q1-Q3])	Vitamin D _ 3_ group (median [Q1-Q3])	Placebo group (median [Q1-Q3])	*p*-value
**PK, nmol/L**	770	0.90 [0.60-1.50]	0.90 [0.60-1.40]	1.00 [0.60-1.50]	.13
**(dp)ucMGP, pmol/L**	771	497.4 [415.5-604.9]	490.8 [420.1-605.5]	502.3 [413.4-603.7]	.84
**%ucOC**	753	63.8 [52.6-78.6]	63.1 [52.2-77.6]	64.6 [52.8-79.9]	.22

### Baseline vitamin K measures and 2-yr changes in bone density and structure

Overall, there were no major differences in 2-yr changes in aBMD at the LS, TH, FN, or whole body between participants by baseline vitamin K status, as determined by levels of PK, (dp)ucMGP, and %ucOC, divided at the median (Table 3). Participants with low vitamin K status by (dp)ucMGP had a small improvement in spine aBMD compared to participants with high vitamin K status (0.54% vs −0.05%, respectively, *p* = .033). This was not corroborated at any other aBMD site or by other vitamin K biomarkers and would not meet criteria for significance after using the Bonferroni correction to adjust for multiple comparisons. We also found no differences in 2-yr changes in TBS between participants with low vs high baseline vitamin K status.

**Table 3:** Baseline vitamin K measures, divided by the median, and 2-yr changes in bone density and structure, adjusted for age, sex, and randomization group.

Bone measure	PK			%ucOC			(dp)ucMGP		
	<Median (0.90 nmol/L; low vitamin K status)	≥Median (high vitamin K status)	*p*-value	≥Median (63.8%; low vitamin K status)	<Median (high vitamin K status)	*p*-value	≥Median (497.4 pmol/L; low vitamin K status)	<Median (high vitamin K status)	*p*-value
**Areal BMD, g/cm** ^**2**^ **, mean (SE)**									
**Spine**									
**Baseline**	1.017 (0.009	1.018 (0.008)		1.029 (0.008)	1.002 (0.008)		1.033 (0.008)	1.003 (0.008)	
**2-yr**	1.017 (0.009)	1.023 (0.008)		1.032 (0.009)	1.003 (0.009)		1.038 (0.009)	1.002 (0.009)	
**% change**	−0.03%	0.46%	.083	0.29%	0.13%	.56	0.54%	−0.05%	.033
**Total hip**									
**Baseline**	0.925 (0.007)	0.933 (0.006)		0.947 (0.006)	0.909 (0.006)		0.942 (0.006)	0.917 (0.006)	
**2-yr**	0.916 (0.007)	0.927 (0.006)		0.939 (0.006)	0.901 (0.007)		0.933 (0.007)	0.911 (0.007)	
**% change**	−1.00%	−0.74%	.18	−0.82%	−0.88%	.90	−0.97%	−0.74%	.18
**Femoral neck**									
**Baseline**	0.761 (0.006)	0.763 (0.006)		0.774 (0.006)	0.747 (0.006)		0.767 (0.006)	0.757 (0.006)	
**2-yr**	0.756 (0.006)	0.760 (0.006)		0.772 (0.006)	0.742 (0.006)		0.763 (0.006)	0.754 (0.006)	
**% change**	−0.65%	−0.34%	.27	−0.27%	−0.67%	.17	−0.63%	−0.32%	.26
**Whole body**									
**Baseline**	1.141 (0.006)	1.144 (0.005)		1.149 (0.006)	1.134 (0.006)		1.144 (0.006)	1.142 (0.006)	
**2-yr**	1.138 (0.006)	1.143 (0.005)		1.147 (0.006)	1.132 (0.006)		1.143 (0.006)	1.139 (0.006)	
**% change**	−0.30%	−0.09%	.22	−0.14%	−0.21%	.69	−0.09%	−0.27%	.26
**Trabecular bone score**									
**Baseline**	1.321 (0.005)	1.317 (0.005)		1.311 (0.005)	1.326 (0.005)		1.304 (0.005)	1.332 (0.005)	
**2-yr**	1.304 (0.006)	1.305 (0.005)		1.299 (0.005)	1.309 (0.005)		1.294 (0.005)	1.315 (0.005)	
**% Change**	−1.28%	−0.89%	.26	−0.94%	−1.23%	.38	−0.80%	−1.30%	.14
**Volumetric bone density and structure**									
**Radius**									
**Total vBMD (4%), mg/cm** ^**3**^									
**Baseline**	374.28 (3.68)	367.26 (3.43)		375.24 (3.38)	363.23 (3.75)		372.53 (3.64)	368.65 (3.52)	
**2-yr**	377.96 (3.95)	375.69 (3.65)		381.76 (3.63)	369.73 (4.02)		378.36 (3.89)	375.26 (3.75)	
**% change**	0.98%	2.29%	.096	1.74%	1.79%	.99	1.56%	1.79%	.78
**Trabecular vBMD (4%), mg/cm** ^**3**^									
**Baseline**	199.89 (2.27)	198.34 (2.11)		201.92 (2.08)	195.65 (2.31)		201.12 (2.23)	197.11 (2.17)	
**2-yr**	200.22 (2.43)	198.74 (2.25)		202.20 (2.23)	195.93 (2.47)		202.06 (2.39)	196.98 (2.31)	
**% change**	0.17%	0.20%	.95	0.14%	0.14%	1.00	0.47%	−0.07%	.31
**Cortical vBMD (33%), mg/cm** ^**3**^									
**Baseline**	1199.3 (1.72)	1195.4 (1.60)		1201.2 (1.59)	1192.3 (1.76)		1196.3 (1.71)	1198.0 (1.65)	
**2-yr**	1200.8 (1.80)	1198.4 (1.67)		1202.2 (1.66)	1195.6 (1.84)		1199.2 (1.79)	1199.8 (1.71)	
**% change**	0.12%	0.25%	.24	0.09%	0.28%	.077	0.24%	0.15%	.43
**Cortical thickness (33%), mm**									
**Baseline**	3.26 (0.02)	3.23 (0.02)		3.30 (0.02)	3.18 (0.02)		3.27 (0.02)	3.22 (0.02)	
**2-yr**	3.21 (0.02)	3.18 (0.02)		3.25 (0.02)	3.12 (0.03)		3.22 (0.02)	3.17 (0.02)	
**% change**	−1.70%	−1.53%	.47	−1.50%	−1.81%	.36	−1.53%	−1.68%	.65
**Tibia**									
**Total vBMD (4%), mg/cm** ^**3**^									
**Baseline**	297.59 (2.47)	295.39 (2.28)		299.74 (2.28)	291.50 (2.53)		299.73 (2.42)	293.25 (2.36)	
**2-yr**	297.70 (2.52)	296.12 (2.33)		300.34 (2.33)	291.72 (2.58)		299.83 (2.47)	293.99 (2.40)	
**% change**	0.04%	0.25%	.29	0.20%	0.07%	.52	0.03%	0.25%	.27
**Trabecular vBMD (4%), mg/cm** ^**3**^									
**Baseline**	248.96 (2.21)	246.93 (2.04)		250.05 (2.04)	244.79 (2.26)		251.55 (2.16)	244.37 (2.10)	
**2-yr**	250.14 (2.26)	247.95 (2.09)		251.41 (2.09)	245.48 (2.31)		252.92 (2.21)	245.21 (2.15)	
**% change**	0.47%	0.41%	.74	0.55%	0.28%	.18	0.54%	0.35%	.28
**Cortical vBMD (38%), mg/cm** ^**3**^									
**Baseline**	1165.6 (1.75)	1163.2 (1.61)		1167.0 (1.61)	1160.4 (1.78)		1163.0 (1.71)	1165.5 (1.67)	
**2-yr**	1168.2 (1.78)	1166.2 (1.64)		1169.3 (1.64)	1163.7 (1.81)		1165.4 (1.74)	1168.8 (1.69)	
**% change**	0.23%	0.26%	.64	0.20%	0.28%	.26	0.20%	0.28%	.28
**Cortical thickness (38%), mm**									
**Baseline**	5.61 (0.04)	5.64 (0.04)		5.68 (0.04)	5.55 (0.04)		5.62 (0.04)	5.63 (0.04)	
**2-yr**	5.57 (0.04)	5.61 (0.04)		5.64 (0.04)	5.51 (0.04)		5.59 (0.04)	5.59 (0.04)	
**% change**	−0.72%	−0.61%	.47	−0.61%	−0.73%	.54	−0.62%	−0.70%	.61
**Bone strength indices**									
Bone strength index, radius (4%), mg*mm									
**Baseline**	46.59 (0.74)	45.16 (0.69)		46.42 (0.69)	44.85 (0.77)		46.24 (0.74)	45.43 (0.71)	
**2-yr**	46.69 (0.78)	46.03 (0.72)		46.89 (0.72)	45.41 (0.80)		46.70 (0.77)	45.99 (0.74)	
**% change**	0.20%	1.93%	.032	1.02%	1.26%	.80	1.00%	1.23%	.79
Bone strength index, tibia (4%), mg*mm									
**Baseline**	105.18 (1.60)	104.57 (1.48)		105.97 (1.48)	102.82 (1.63)		107.28 (1.57)	102.55 (1.53)	
**2-yr**	105.54 (1.63)	105.00 (1.50)		106.62 (1.50)	102.91 (1.66)		107.46 (1.59)	103.14 (1.55)	
**% change**	0.34%	0.40%	.86	0.61%	0.08%	.13	0.16%	0.57%	.25
**Polar stress strength index, radius (33%), mm** ^**3**^									
**Baseline**	282.00 (3.56)	287.27 (3.31)		283.36 (3.32)	286.70 (3.67)		286.23 (3.52)	283.50 (3.41)	
**2-yr**	283.26 (3.61)	287.31 (3.36)		284.00 (3.37)	287.21 (3.72)		286.79 (3.58)	284.14 (3.45)	
**% change**	0.44%	0.01%	.23	0.23%	0.18%	.90	0.19%	0.23%	.94
**Polar stress strength index, tibia (38%), mm** ^**3**^									
**Baseline**	1875.0 (19.96)	1875.9 (18.36)		1851.4 (18.29)	1899.3 (20.26)		1860.3 (19.52)	1890.0 (19.04)	
**2-yr**	1880.6 (19.92)	1885.8 (18.32)		1856.7 (18.26)	1910.6 (20.23)		1865.4 (19.48)	1900.4 (18.98)	
**% change**	0.30%	0.53%	.27	0.28%	0.60%	.13	0.28%	0.55%	.19

We also performed subgroup analyses by sex in exploratory analyses (Table S1). There were no differences in 2-yr changes in aBMD at the LS, TH, FN, or whole body or TBS in men or women by baseline vitamin K measures, as assessed by PK levels or %ucOC. Women with low vitamin K status by (dp)ucMGP had less bone loss at the LS compared to women with high vitamin K status (−0.02% vs −1.07%, respectively, *p* = .019). This was not seen in men, and there was no significant interaction by sex. Again, this is likely a chance finding as this was not corroborated at any other aBMD site or by other vitamin K biomarkers.

Baseline vitamin K status, as assessed by these biomarkers, was also not associated with 2-yr changes in bone structure and architecture as at the radius and tibia as measured by pQCT (Table 3). There were no differences in 2-yr changes in total vBMD, trabecular vBMD, cortical vBMD, or cortical thickness at the radius or at the tibia between participants with low vs high vitamin K status. Participants with PK levels above the median did have a slight increase in BSI at the radius compared to those with PK levels below the median (1.93% vs 0.20%, respectively, *p* = .032). However, this was not supported by benefits at other sites, with other strength measures, or by other vitamin K measures.

In exploratory analyses, total OC, a marker of bone formation, increased in participants with low vitamin K status as indicated by %ucOC, compared to those with high vitamin K status (*p* < .001) (Table S2). However, there were no differences observed with other BTMs or by vitamin K status by other biomarkers.

We then investigated whether there was a low threshold of vitamin K sufficiency for bone outcomes by using a PK cut off of ≤0.5 nmol/L (Table 4 and Table S3). Participants with PK level <0.5 nmol/L had slightly more bone loss compared to those with higher PK levels at the TH (−1.22% and −0.76%, respectively, *p* = .048) and at the whole body (−0.65% and −0.06%, respectively, *p* = .003). However, there were no differences in 2-yr changes in aBMD at spine, FN, TBS, vBMD, cortical thickness, and bone strength indices between those with low PK levels and higher PK levels. In exploratory analyses, P1NP increased more in participants with low PK levels than those with higher PK levels, but no differences were seen in other BTMs.

**Table 4:** Baseline vitamin K measures, divided by clinical significance, and 2-yr changes in bone density, adjusted for age, sex, and randomization group.

Bone measure	PK					(dp)ucMGP				
	≤0.5 nmol/L (low vitamin K status)		>0.5 nmol/L (higher vitamin K status)		*p*-value	>300 pmol/L (lower vitamin K status)		≤300 pmol/L (high vitamin K status)		*p*-value
	*n*	Mean (SE)	*n*	Mean (SE)		*n*	Mean (SE)	*n*	Mean (SE)	
**Areal BMD, g/cm** ^**2**^ **, mean (SE)**										
**Spine**										
**Baseline**	155	1.021 (0.012)	564	1.017 (0.006)		696	1.018 (0.006)	24	0.995 (0.032)	
**2-yr**	131	1.023 (0.013)	503	1.019 (0.007)		613	1.022 (0.006)	21	0.983 (0.034)	
**% Change**		0.21%		0.26%	.88		0.30%		−1.27%	.049
**Total hip**										
**Baseline**	166	0.923 (0.010)	598	0.931 (0.005)		741	0.930 (0.005)	24	0.925 (0.026)	
**2-yr**	145	0.912 (0.010)	534	0.924 (0.0050		657	0.922 (0.005)	22	0.917 (0.026)	
**% Change**		−1.22%		−0.76%	.048		−0.86%		−0.83%	.96
**Femoral neck**										
**Baseline**	164	0.760 (0.009)	596	0.763 (0.005)		737	0.762 (0.004)	24	0.783 (0.024)	
**2-yr**	143	0.753 (0.009)	532	0.76 (0.005)		653	0.758 (0.004)	22	0.786 (0.024)	
**% Change**		−0.92%		−0.36%	.099		−0.50%		0.31%	.30
**Whole body**										
**Baseline**	159	1.143 (0.008)	580	1.142 (0.004)		716	1.143 (0.004)	24	1.144 (0.022)	
**2-yr**	133	1.136 (0.009)	495	1.142 (0.004)		606	1.141 (0.004)	22	1.142 (0.022)	
**% Change**		−0.65%		−0.06%	.003		−0.18%		−0.22%	.94

We performed similar analyses to examine whether there was a high threshold of vitamin K sufficiency by using a (dp)ucMGP cut off of ≤300 pmol/L (Table 4 and Table S3). This was limited as only 24 participants reached this threshold. Participants with high vitamin K status had greater bone loss at the spine than participants with lower vitamin K status at 2-yr follow-up (−1.27% vs 0.30%, respectively, *p* = .049). However, there were no differences in 2-yr changes in the aBMD at the hip and whole body, vBMD, or other architectural bone parameters. In exploratory analyses, P1NP decreased more in the few participants with robust vitamin K activity, compared to others with less vitamin K activity, but no differences were seen in other BTMs.

Finally, in exploratory analyses, we also compared the lowest quartile to the highest quartile of vitamin K status and their association with 2-yr changes in bone density and structure (Table S4). Overall, there was no consistent benefit for participants in the highest quartile of vitamin K status compared to those in the lowest quartile. Participants in the highest quartile of PK levels had greater improvements in total vBMD and BSI at the radius compared to those in the lowest quartile (*p* = .017 and 0.025, respectively). However, participants with the highest vitamin K status by (dp)ucMGP had worse outcomes at the spine aBMD and trabecular vBMD at the radius (*p* = .005 and 0.013, respectively), but greater polar SSI at the tibia (*p* = .007).

### Baseline vitamin K and vitamin D measures and 2-yr changes in bone density and structure

We also investigated whether the combination of low vitamin K and D status was associated with increased bone loss and deterioration of bone structure and architecture over 2 yr, compared to participants with high vitamin K and D status, both divided at the median. There were no differences in 2-yr changes in aBMD at the spine, TH, FN or whole body between participants with low vitamin K and D status and high vitamin K and D status (Table 5).

**Table 5:** Baseline vitamin K and D measures, divided at the median, and 2-yr changes in bone density and structure, adjusted for age, sex, and randomization group.

Bone Measure	PK and 25(OH)D					%ucOC and 25(OH)D					(dp)ucMGP and 25(OH)D				
	Low vitamin K (PK <0.90 nmol/L) and low vitamin D (25[OH]D < 70 nmol/L) status		High vitamin K (PK ≥0.90 nmol/L) and high vitamin D (25[OH]D ≥ 70 nmol/L) status		*p*-value	Low vitamin K (%ucOC ≥63.8%) and low vitamin D (25[OH]D < 70 nmol/L) status		High vitamin K (%ucOC <63.8%) and high vitamin D (25[OH]D ≥ 70 nmol/L) status		*p*-value	Low vitamin K ([dp]ucMGP ≥497.4 pmol/L) and low vitamin D (25[OH]D < 70 nmol/L) status		High vitamin K ([dp]ucMGP <497.4 pmol/L) and high vitamin D (25[OH]D ≥ 70 nmol/L) status		*p*-value
	*n*	Mean (SE)	*n*	Mean (SE)		*n*	Mean (SE)	*n*	Mean (SE)		*n*	Mean (SE)	*n*	Mean (SE)	
**Areal BMD, g/cm** ^**2**^ **, as assessed by DXA**															
**Spine**															
**Baseline**	173	1.025 (0.012)	209	1.012 (0.010)		180	1.027 (0.011)	174	0.978 (0.011)		186	1.034 (0.012)	182	0.991 (0.012)	
**2-yr**	149	1.024 (0.012)	186	1.016 (0.011)		157	1.032 (0.012)	155	0.982 (0.012)		159	1.040 (0.012)	161	0.991 (0.012)	
**% Change**		−0.08%		0.40%	.21		0.44%		0.35%	.78		0.60%		0.03%	.12
**Total hip**															
**Baseline**	183	0.936 (0.009)	228	0.934 (0.008)		187	0.944 (0.009)	188	0.897 (0.009)		195	0.944 (0.009)	200	0.915 (0.009)	
**2-yr**	160	0.928 (0.010)	203	0.926 (0.009)		165	0.937 (0.009)	167	0.888 (0.009)		169	0.936 (0.009)	178	0.907 (0.009)	
**% Change**		−0.87%		−0.83%	.90		−0.80%		−1.01%	.54		−0.83%		−0.81%	.85
**Femoral neck**															
**Baseline**	181	0.771 (0.009)	228	0.765 (0.008)		184	0.770 (0.008)	188	0.738 (0.008)		194	0.771 (0.009)	200	0.756 (0.008)	
**2-yr**	158	0.767 (0.009)	203	0.761 (0.008)		162	0.769 (0.008)	167	0.730 (0.008)		168	0.767 (0.009)	178	0.751 (0.009)	
**% Change**		−0.52%		−0.60%	.83		−0.22%		−0.97%	.063		−0.47%		−0.59%	.80
**Whole body**															
**Baseline**	177	1.144 (0.008)	219	1.144 (0.007)		182	1.145 (0.008)	181	1.128 (0.008)		188	1.140 (0.008)	194	1.138 (0.008)	
**2-yr**	145	1.143 (0.008)	187	1.142 (0.007)		148	1.146 (0.008)	158	1.125 (0.008)		153	1.141 (0.008)	168	1.135 (0.008)	
**% Change**		−0.10%		−0.14%	.88		0.09%		−0.23%	.17		0.10%		−0.29%	.091
**Trabecular bone score**															
**Baseline**	159	1.323 (0.008)	201	1.325 (0.007)		159	1.304 (0.007)	169	1.323 (0.007)		160	1.299 (0.008)	178	1.329 (0.007)	
**2-yr**	138	1.302 (0.008)	176	1.310 (0.007)		141	1.294 (0.008)	148	1.307 (0.008)		137	1.290 (0.008)	156	1.313 (0.008)	
**% Change**		−1.56%		−1.09%	.32		−0.81%		−1.21%	.38		−0.69%		−1.21%	.28
**Volumetric BMD, as assess by pQCT**															
**Radius**															
**Total vBMD (4%), mg/cm** ^**3**^															
**Baseline**	161	378.57 (5.15)	188	368.40 (4.70)		172	373.36 (4.97)	145	357.20 (5.43)		158	371.42 (5.18)	175	365.76 (4.97)	
**2-yr**	136	386.16 (5.47)	166	377.04 (4.96)		147	382.10 (5.33)	125	363.49 (5.82)		132	379.43 (5.53)	151	371.26 (5.28)	
**% Change**		2.00%		2.35%	.78		2.34%		1.76%	.56		2.16%		1.50%	.54
**Trabecular vBMD (4%), mg/cm** ^**3**^															
**Baseline**	161	201.65 (3.12)	188	199.14 (2.84)		172	200.21 (3.05)	145	192.51 (3.34)		158	200.86 (3.15)	175	196.79 (3.03)	
**2-yr**	136	201.68 (3.38)	166	198.55 (3.08)		147	199.98 (3.28)	125	191.28 (3.58)		132	201.77 (3.45)	151	195.82 (3.31)	
**% Change**		0.01%		−0.29%	.68		−0.11%		−0.64%	.54		0.46%		−0.49%	.22
**Cortical vBMD (33%), mg/cm** ^**3**^															
**Baseline**	154	1204.3 (2.41)	179	1194.3 (2.20)		165	1202.9 (2.37)	138	1187.2 (2.58)		155	1197.8 (2.38)	168	1193.6 (2.27)	
**2-yr**	126	1205.0 (2.60)	160	1198.4 (2.35)		133	1203.1 (2.41)	120	1191.6 (2.60)		113	1198.3 (2.51)	146	1195.5 (2.35)	
**% Change**		0.05%		0.34%	.071		0.02%		0.37%	.028		0.05%		0.16%	.46
**Cortical thickness (33%), mm**															
**Baseline**	154	3.27 (0.04)	179	3.23 (0.03)		165	3.29 (0.03)	138	3.17 (0.03)		155	3.27 (0.03)	168	3.22 (0.03)	
**2-yr**	126	3.22 (0.04)	160	3.18 (0.03)		133	3.24 (0.03)	120	3.11 (0.03)		113	3.22 (0.03)	146	3.16 (0.03)	
**% Change**		−1.57%		−1.59%	1.00		−1.45%		−1.90%	.32		−1.42%		−1.75%	.45
**Tibia**															
**Total vBMD (4%), mg/cm** ^**3**^															
**Baseline**	162	300.16 (3.46)	195	293.04 (3.12)		177	298.80 (3.19)	149	284.06 (3.47)		162	299.98 (3.22)	178	289.31 (3.10)	
**2-yr**	139	300.56 (3.53)	173	293.75 (3.19)		151	299.21 (3.21)	132	284.06 (3.49)		135	300.51 (3.31)	159	290.20 (3.19)	
**% Change**		0.13%		0.24%	.71		0.14%		0.00%	.57		0.17%		0.31%	.64
**Trabecular vBMD (4%), mg/cm** ^**3**^															
**Baseline**	162	250.13 (3.08)	195	245.51 (2.79)		177	247.91 (2.84)	149	238.82 (3.09)		162	251.23 (2.84)	178	242.48 (2.74)	
**2-yr**	139	251.58 (3.15)	173	246.25 (2.84)		151	249.37 (2.89)	132	238.99 (3.14)		135	253.08 (2.91)	159	243.14 (2.80)	
**% Change**		0.58%		0.30%	.27		0.59%		0.07%	.052		0.74%		0.27%	.026
**Cortical vBMD (38%), mg/cm** ^**3**^															
**Baseline**	161	1167.6 (2.54)	193	1160.3 (2.29)		174	1168.4 (2.29)	148	1156.0 (2.49)		163	1163.9 (2.40)	175	1161.5 (2.32)	
**2-yr**	139	1170.5 (2.58)	173	1162.9 (2.33)		151	1171.1 (2.31)	133	1158.9 (2.50)		135	1166.9 (2.43)	159	1164.6 (2.34)	
**% Change**		0.25%		0.22%	.73		0.23%		0.25%	.89		0.26%		0.26%	.98
**Cortical thickness (38%), mm**															
**Baseline**	161	5.58 (0.06)	193	5.71 (0.05)		174	5.60 (0.05)	148	5.58 (0.06)		163	5.59 (0.06)	175	5.70 (0.05)	
**2-yr**	139	5.54 (0.06)	173	5.66 (0.05)		151	5.57 (0.06)	133	5.53 (0.06)		135	5.56 (0.06)	159	5.65 (0.05)	
**% Change**		−0.73%		−0.75%	.87		−0.60%		−0.89%	.21		−0.54%		−0.78%	.22
**Bone strength indices**															
Bone strength index, radius (4%), mg*mm															
**Baseline**	161	47.79 (1.06)	188	45.74 (0.96)		172	46.50 (1.01)	145	44.12 (1.11)		158	46.02 (1.06)	175	45.04 (1.02)	
**2-yr**	136	48.34 (1.10)	166	46.41 (1.01)		147	47.12 (1.03)	125	44.32 (1.13)		132	46.78 (1.11)	151	45.29 (1.07)	
**% Change**		1.15%		1.47%	.80		1.33%		0.45%	.39		1.66%		0.55%	.33
Bone strength index, tibia (4%), mg*mm															
**Baseline**	162	106.80 (2.23)	195	104.68 (2.02)		177	105.33 (2.02)	149	99.39 (2.20)		162	106.05 (2.03)	178	100.45 (1.96)	
**2-yr**	139	107.27 (2.26)	173	104.93 (2.05)		151	105.82 (2.03)	132	99.08 (2.21)		135	106.49 (2.06)	159	100.98 (1.99)	
**% Change**		0.44%		0.24%	.65		0.47%		−0.31%	.11		0.42%		0.53%	.83
**Polar stress strength index, radius (33%), mm** ^**3**^															
**Baseline**	154	285.71 (4.73)	179	291.17 (4.30)		165	282.52 (4.81)	138	287.23 (5.23)		155	283.05 (4.55)	168	284.05 (4.34)	
**2-yr**	126	287.73 (4.81)	160	289.57 (4.36)		133	284.66 (4.92)	120	286.45 (5.34)		113	284.49 (4.65)	146	282.93 (4.43)	
**% Change**		0.71%		−0.55%	.013		0.76%		−0.27%	.048		0.51%		−0.40%	.088
**Polar stress strength index, tibia (38%), mm** ^**3**^															
**Baseline**	161	1897.0 (25.74)	193	1901.8 (23.27)		174	1848.2 (24.68)	148	1899.7 (26.85)		163	1855.8 (26.85)	175	1890.5 (25.97)	
**2-yr**	139	1901.3 (25.50)	173	1909.3 (23.04)		151	1853.9 (24.72)	133	1909.2 (26.88)		135	1863.1 (26.59)	159	1900.3 (25.68)	
**% Change**		0.23%		0.40%	.57		0.31%		0.50%	.49		0.39%		0.52%	.63

Overall, there were also no major differences in 2-yr changes in bone architecture by TBS and pQCT as well as bone strength indices by pQCT (Table 5). Participants with high vitamin K, as assessed by %ucOC, and high vitamin D status had slightly greater gains in cortical vBMD at the radius, compared to those with low vitamin K and D status (0.37% vs 0.02%, respectively, *p* = .028). However, participants with high vitamin K, as assessed by (dp)ucMGP, and high vitamin D status had less gains in trabecular vBMD at the tibia, compared to those with low vitamin K and D status (0.27% vs 0.74%, respectively, *p* = .026). Participants with high vitamin K, as assessed by PK and %ucOC, and high vitamin D status also had slightly worsened polar SSI at the radius compared to those with low vitamin K and vitamin D status (−0.55% vs 0.71%, respectively, *p* = .01, for PK and −0.27% vs 0.76%, respectively, *p* = .048, for %ucOC). These results would not meet criterion for significance after using the Bonferroni correction to adjust for multiple comparisons.

In exploratory analyses, total OC levels had increased in participants with low vitamin K and D status, as assessed by %uOC and 25OHD, while total OC levels slightly declined in those with high K and D status (11.66% vs −1.17%, respectively, *p* = .001) (Table S5). Participants with low vitamin K and D status, as assessed by PK and 25OHD, had a decline in CTX levels, while those with high K and D status had a rise in CTX levels (−3.21% vs 5.50%, respectively, *p* = .034).

### Effect modification of supplemental vitamin D on changes in aBMD and TBS by baseline vitamin K measures

The effects of supplemental vitamin D_3_ at a dose of 2000 units/d on 2-yr changes in aBMD at spine, TH, FN, and whole body were not modified by baseline measures of PK, %ucOC, and (dp)ucMGP measures, divided at the median (Table 6). In exploratory analyses, baseline vitamin K status also did not affect response of supplementation vitamin D on 2-yr changes in TBS or BTMs (Table S6). Measures of vitamin K status at year 2 also did not modify effects of supplemental vitamin D_3_ on changes in aBMD, TBS, or BTMs in exploratory analyses (Table S7).

**Table 6:** Effect modification of supplemental vitamin D on 2-year changes in areal BMD by baseline vitamin K measures, adjusted for age, sex, and randomization group.

Bone measure		Vitamin D Group			Placebo Group			*p*, treatment effect	*p* for interaction
		*N*	Difference (SE)	*p*-value	*N*	Difference (SE)	*p*-value		
**PK**	Spine, g/cm^2^								.32
	<Median (0.90 nmol/L)	184	0.002 (0.003)	.43	162	−0.003 (0.003)	.34	.22	
	≥Median	204	0.004 (0.003)	.12	220	0.005 (0.003)	.07	.87	
	Total hip, g/cm^2^								.74
	<Median (0.90 nmol/L)	184	−0.008 (0.002)	<.001	162	−0.011 (0.002)	<.001	.37	
	≥Median	204	−0.006 (0.002)	<.001	220	−0.008 (0.002)	<.001	.52	
	Femoral neck, g/cm^2^								.51
	<Median (0.90 nmol/L)	184	−0.003 (0.002)	.25	162	−0.008 (0.002)	.002	.15	
	≥Median	204	−0.002 (0.002)	.44	220	−0.004 (0.002)	.060	.47	
	Whole body, g/cm^2^								.44
	<Median (0.90 nmol/L)	184	−0.003 (0.002)	.16	162	−0.004 (0.002)	.082	.79	
	≥Median	204	−0.002 (0.002)	.22	220	0.000 (0.002)	.94	.40	
**%ucOC**	Spine, g/cm^2^								.87
	≥Median (63.8%)	183	0.003 (0.003)	.31	194	0.003 (0.003)	.31	.99	
	<Median	195	0.002 (0.003)	.50	181	0.001 (0.003)	.78	.80	
	Total hip, g/cm^2^								.25
	≥Median (63.8%)	183	−0.006 (0.002)	<.001	194	−0.010 (0.002)	<.001	.14	
	<Median	195	−0.008 (0.002)	<.001	181	−0.008 (0.002)	<.001	.85	
	Femoral neck, g/cm^2^								.77
	≥Median (63.8%)	183	−0.001 (0.002)	.66	194	−0.003 (0.002)	.14	.48	
	<Median	195	−0.003 (0.002)	.11	181	−0.007 (0.002)	.002	.27	
	Whole body, g/cm^2^								.36
	≥Median (63.8%)	183	−0.001 (0.002)	.60	194	−0.002 (0.002)	.26	.70	
	<Median	195	−0.004 (0.002)	.043	181	−0.001 (0.002)	.49	.36	
**(dp)ucMGP**	Spine, g/cm^2^								.81
	≥Median (497.4 pmol/L)	188	0.007 (0.003)	.021	197	0.004 (0.003)	.16	.55	
	<Median	200	0.000 (0.003)	.97	186	−0.001 (0.003)	.73	.78	
	Total hip, g/cm^2^								.86
	≥Median (497.4 pmol/L)	188	−0.008 (0.002)	<.001	197	−0.010 (0.002)	<.001	.62	
	<Median	200	−0.006 (0.001)	<.001	186	−0.008 (0.002)	<.001	.34	
	Femoral neck, g/cm^2^								.40
	≥Median (497.4 pmol/L)	188	−0.004 (0.002)	.068	197	−0.006 (0.002)	.015	.69	
	<Median	200	0.000 (0.002)	.98	186	−0.005 (0.002)	.014	.078	
	Whole body, g/cm^2^								.86
	≥Median (497.4 pmol/L)	188	−0.001 (0.002)	.55	197	−0.001 (0.002)	.68	.88	
	<Median	200	−0.004 (0.002)	.036	186	−0.003 (0.002)	.15	.66	

## Discussion

In this ancillary study titled “VITAL: Interrelationship of Vitamin D and Vitamin K on Bone,” low vitamin K status, alone or in combination with low baseline vitamin D level, in a generally healthy population not selected for osteoporosis was not associated greater bone loss at the spine, hip, or whole body; worsening TBS; or increased deterioration of trabecular or cortical measures at the radius and tibia, compared to high vitamin K status. Furthermore, vitamin K status did not modify effects of vitamin D supplementation on 2-yr changes in aBMD or TBS.

Despite the lack of robust evidence, vitamin K is increasingly being advertised to improve bone health and is often included with vitamin D supplementation. Observational studies have found associations of low vitamin K intake or biomarkers with low bone density.^5–8^ A 2019 systematic review of 22 trials in postmenopausal or osteoporotic women found that supplemental vitamin K (either K1 or K2 in the form of menaquinone-4 or menaquinone-7) did improve bone density slightly at the spine (mean difference of 1.63% [95% CI: 0.10-3.16])) and femur (mean difference of 0.45% [95% CI: 0.05-0.85]) at 2-yr follow-up.^26^^,^^27^ These modest improvements in BMD from vitamin K supplementation were less than the threshold needed to reduce fracture risk, as determined by the Strategy to Advance BMD as a Regulatory Endpoint (SABRE) project.^28^ Indeed, the meta-analyses found that vitamin K supplementation reduced odds of any clinical fracture by 24%, but not vertebral fractures, and statistical significance was lost when analyses were restricted to low risk of bias trials.^26^^,^^27^

More recent small, randomized controlled trials with concurrent supplemental vitamin K and D have also not found a benefit on bone health measures. Moore et al. randomized 105 postmenopausal women on oral bisphosphonates to vitamin K1, MK-4, and placebo; all participants were on vitamin D supplements, and mean baseline 25OHD level was 32 ng/mL.^29^ After 18 mo of treatment, changes in bone density at the LS, TH, and FN did not differ in the women who did or did not receive a form of vitamin K. In another randomized controlled trial, 142 postmenopausal women with osteopenia were randomized to MK-7 or placebo and all received vitamin D_3_ 1500 IU/d and calcium 800 mg/d.^30^ Compared to placebo, the women in the MK-7 group had decreases in ucOC. However, after 3 yr of treatment, there were no differences in BTMs, aBMD, TBS, or microarchitecture as assessed by HR-pQCT.

Finally, concurrent supplementation with vitamin K and D does not seem to have additive or synergistic effects on bone density beyond supplementation with vitamin K or D alone. A Chinese trial randomized 311 adults aged 50-75 yr with T-score <−1.0 into 4 groups: MK-7 50 mcg/d, MK-7 90 mcg/d, MK-7 90 mcg/d + vitamin D_3_ 400 IU/d + calcium 500 mg/d, and placebo.^31^ In all groups receiving vitamin K, carboxylated OC/ucOC ratio increased. However, there was no difference in bone density among treatment groups after 1 yr. In subgroup analyses, the higher dose of MK-7 did reduce FN bone loss in women, without any additional effects from calcium and vitamin D co-supplementation.

Similar to how the perceived threshold for bone benefits of vitamin D seem to be declining over the years, the threshold for vitamin K sufficiency may also be low. Although we saw no benefit of vitamin K status, when divided at the median, participants with very low PK levels (≤0.05 nmoL/L) did have greater bone loss at the whole body, compared to participants with higher PK levels. In the large prospective Nurses’ Health Study with 72 327 women and 270 hip fractures, there was no association between daily vitamin K intake, divided by quintiles, and hip fracture risk.^10^ When only the first quintile of vitamin K intake (<109 mcg/d) was compared to the 4 higher quintiles, there was a 30% increase in hip fracture risk among the participants with the lowest vitamin K intake. This would be consistent with the adequate intake of 90 mcg/d for women as set by the Institute of Medicine.^32^

For vitamin D, there is an emerging concern that too much vitamin D may result in bone loss due to promotion of osteoclastic activity. Other clinical trials have noted a positive correlation in supplemental vitamin D dose and CTX levels.^33^^,^^34^ In the randomized clinical trial with vitamin D doses of 400 IU/d, 4000 IU/d, or 10 000 IU/d by Burt et al., supplementation with vitamin D doses of 4000 IU/d and 10 000 IU/d for 3 yr resulted in greater bone loss at the radius as assessed by HR-pQCT compared to the dose of 400 IU/d.^33^ In this clinical trial, the 4000 IU group reached a mean 25OHD level of 132.2 nmol/L and the 10 000 IU group reached a peak level of 200.4 nmol/L. The 10 000 IU group had the highest levels of CTX. High-dose vitamin D supplementation may result in stimulation of osteoclastogenesis and differentiation as calcitriol has been found to be a potent inducer of receptor activator of NF-κB ligand in vitro.^33^^,^^35^ In the bone health cohort in VITAL, the active vitamin D group had a mean total 25OHD level of 98.6 nmol/L at 2-yr follow-up. Although our mean 25OHD level was within the range recommended by the Institute of Medicine,^36^ participants with high vitamin D and high vitamin K status did have greater increases in CTX levels than participants with low vitamin D and K status in our study.

This ancillary study has several strengths. This is the first randomized controlled trial to test whether baseline vitamin K status influences effects of supplemental vitamin D on bone density and architecture in vitamin D replete healthy adults. Vitamin K status was assessed by 3 different sensitive biomarkers, and standards from the Center for Diseases Control were used to analyze 25OHD levels. However, we did not have information on use of vitamin K supplementation (alone or within multivitamin or calcium and vitamin D supplements) or sufficient dietary information to assess vitamin K intake. Our study was not designed to randomize participants to both vitamin K and vitamin D supplements. Bone parameters were measured at baseline and 2-yr follow up, so our findings do not preclude the possibility that longer follow-up may be necessary to observe a benefit of vitamin K status on bone density and architecture. By design, participants in the bone health sub-cohort did not have osteoporosis, unless they wished to defer osteoporosis treatment, as we did not want the use of osteoporosis medications to confound bone measures. Thus, these findings are not generalizable to patients with osteoporosis, who may benefit the most.

Higher vitamin K status, alone or together with higher vitamin D status, at baseline did not protect against bone loss and deterioration of bone structure and architecture. Furthermore, higher baseline vitamin K status did not modify or augment the response to vitamin D supplementation in improving these bone measures, compared to placebo. The results from this ancillary VITAL study indicate that vitamin K does not play a large role in changes in bone density and architecture in a generally healthy population.

## Supplementary Material

Vitamin_K_BMD_cohort_supp_tables_JBMR_Plus_5-14-26_clean_ziag110

## Data Availability

Data described in the manuscript, code book, and analytic code will be made available upon request pending application and approval.
