# Supplementary material for: Investigating the interrelationship of vitamin K and vitamin D status on bone density: results from the VITamin D and OmegA-3 TriaL
Source: JBMR Plus. 2026 Jul 9;10(8):ziag110. doi: 10.1093/jbmrpl/ziag110 (PMC13411269; doi:10.1093/jbmrpl/ziag110)
Supplement: Vitamin_K_BMD_cohort_supp_tables_JBMR_Plus_5-14-26_clean_ziag110 [file vitamin_k_bmd_cohort_supp_tables_jbmr_plus_5-14-26_clean_ziag110.docx]

Supplemental Table 1. Baseline Vitamin K Measures, divided by the median, and 2-year Changes in Areal Bone Density and Structure, According to Sex

| **Bone Measure** | **PK** | | | | | | |
| --- | --- | --- | --- | --- | --- | --- | --- |
|  | **Men** | | | **Women** | | | **p for interaction** |
|  | **<median (0.90 nmol/L; low vitamin K status)** | **≥median**  **(high vitamin K status)** | **p-value** | **<median (0.90 nmol/L; low vitamin K status)** | **≥median**  **(high vitamin K status)** | **p-value** |  |
| Areal Bone Mineral Density, g/cm^2^, mean (SE) | | | | | | | |
| Spine | | | | | | | |
| Baseline | 1.081 (0.012) | 1.072 (0.011) |  | 0.953 (0.012) | 0.964 (0.011) |  |  |
| 2-year | 1.086 (0.013) | 1.084 (0.012) |  | 0.946 (0.013) | 0.960 (0.011) |  |  |
| % change | 0.42% | 1.07% | 0.074 | -0.64% | -0.38% | 0.56 | 0.45 |
| Total Hip | | | | | | | |
| Baseline | 0.996 (0.010) | 1.016 (0.009) |  | 0.854 (0.009) | 0.851 (0.008) |  |  |
| 2-year | 0.992 (0.010) | 1.014 (0.009) |  | 0.839 (0.009) | 0.838 (0.008) |  |  |
| % change | -0.44% | -0.22% | 0.33 | -1.79% | -1.49% | 0.32 | 0.86 |
| Femoral Neck | | | | | | | |
| Baseline | 0.809 (0.009) | 0.819 (0.008) |  | 0.714 (0.008) | 0.706 (0.007) |  |  |
| 2-year | 0.806 (0.009) | 0.820 (0.009) |  | 0.707 (0.008) | 0.699 (0.007) |  |  |
| % change | -0.35% | 0.14% | 0.17 | -1.04% | -1.01% | 0.93 | 0.40 |
| Whole Body | | | | | | | |
| Baseline | 1.214 (0.008) | 1.224 (0.008) |  | 1.069 (0.008) | 1.064 (0.007) |  |  |
| 2-year | 1.213 (0.008) | 1.228 (0.008) |  | 1.062 (0.008) | 1.057 (0.007) |  |  |
| % change | -0.03% | 0.30% | 0.081 | -0.71% | -0.07% | 0.99 | 0.26 |
| Trabecular Bone Score | | | | | | | |
| Baseline | 1.342 (0.008) | 1.330 (0.007) |  | 1.299 (0.007) | 1.304 (0.006) |  |  |
| 2-year | 1.328 (0.008) | 1.323 (0.007) |  | 1.277 (0.008) | 1.286 (0.007) |  |  |
| % change | -1.00% | -0.50% | 0.30 | -1.67% | -1.40% | 0.59 | 0.72 |
|  | **%ucOC** | | | | | | |
|  | **Men** | | | **Women** | | | **p for interaction** |
|  | **≥median**  **(63.8%; low vitamin K status)** | **<median**  **(high vitamin K status)** | **p-value** | **≥median**  **(63.8%; low vitamin K status)** | **<median**  **(high vitamin K status)** | **p-value** |  |
| Areal Bone Mineral Density, g/cm^2^, mean (SE) | | | | | | | |
| Spine | | | | | | | |
| Baseline | 1.086 (0.012) | 1.064 (0.011) |  | 0.971 (0.011) | 0.938 (0.012) |  |  |
| 2-year | 1.097 (0.013) | 1.070 (0.012) |  | 0.966 (0.011) | 0.932 (0.013) |  |  |
| % change | 0.99% | 0.57% | 0.23 | -0.50% | -0.64% | 0.77 | 0.54 |
| Total Hip | | | | | | | |
| Baseline | 1.028 (0.010) | 0.989 (0.009) |  | 0.867 (0.008) | 0.828 (0.009) |  |  |
| 2-year | 1.025 (0.010) | 0.985 (0.009) |  | 0.853 (0.008) | 0.814 (0.009) |  |  |
| % change | -0.21% | -0.41% | 0.40 | -1.58% | -1.70% | 0.89 | 0.67 |
| Femoral Neck | | | | | | | |
| Baseline | 0.828 (0.009) | 0.803 (0.008) |  | 0.721 (0.007) | 0.692 (0.008) |  |  |
| 2-year | 0.830 (0.009) | 0.800 (0.009) |  | 0.714 (0.007) | 0.684 (0.008) |  |  |
| % change | -0.29% | -0.37% | 0.066 | -0.94% | -1.16% | 0.70 | 0.34 |
| Whole Body | | | | | | | |
| Baseline | 1.226 (0.008) | 1.213 (0.008) |  | 1.071 (0.007) | 1.056 (0.008) |  |  |
| 2-year | 1.228 (0.008) | 1.215 (0.008) |  | 1.066 (0.007) | 1.046 (0.008) |  |  |
| % change | 0.15% | 0.16% | 0.96 | -0.52% | -0.95% | 0.14 | 0.24 |
| Trabecular Bone Score | | | | | | | |
| Baseline | 1.324 (0.008) | 1.343 (0.007) |  | 1.299 (0.007) | 1.307 (0.007) |  |  |
| 2-year | 1.316 (0.008) | 1.332 (0.007) |  | 1.282 (0.007) | 1.282 (0.008) |  |  |
| % change | -0.62% | -0.86% | 0.62 | -1.29% | -1.88% | 0.22 | 0.64 |
|  | **(dp)ucMGP** | | | | | | |
|  | **Men** | | | **Women** | | | **p for interaction** |
|  | **≥median**  **(497.4 pmol/L; low vitamin K status)** | **<median**  **(high vitamin K status)** | **p-value** | **≥median**  **(497.4 pmol/L; low vitamin K status)** | **<median**  **(high vitamin K status)** | **p-value** |  |
| Areal Bone Mineral Density, g/cm^2^, mean (SE) | | | | | | | |
| Spine | | | | | | | |
| Baseline | 1.081 (0.012) | 1.072 (0.011) |  | 0.984 (0.011) | 0.930 (0.012) |  |  |
| 2-year | 1.092 (0.013) | 1.078 (0.012) |  | 0.984 (0.011) | 0.920 (0.012) |  |  |
| % change | 1.02% | 0.58% | 0.21 | -0.02% | -1.07% | 0.019 | 0.38 |
| Total Hip | | | | | | | |
| Baseline | 1.009 (0.010) | 1.005 (0.009) |  | 0.875 (0.008) | 0.827 (0.009) |  |  |
| 2-year | 1.004 (0.010) | 1.004 (0.009) |  | 0.862 (0.008) | 0.813 (0.009) |  |  |
| % change | -0.52% | -0.14% | 0.087 | -1.53% | -1.72% | 0.75 | 0.17 |
| Femoral Neck | | | | | | | |
| Baseline | 0.811 (0.009) | 0.817 (0.009) |  | 0.724 (0.007) | 0.695 (0.008) |  |  |
| 2-year | 0.809 (0.009) | 0.818 (0.009) |  | 0.716 (0.007) | 0.688 (0.008) |  |  |
| % change | -0.29% | 0.11% | 0.26 | -1.04% | -1.01% | 0.87 | 0.51 |
| Whole Body | | | | | | | |
| Baseline | 1.212 (0.008) | 1.226 (0.008) |  | 1.075 (0.007) | 1.057 (0.008) |  |  |
| 2-year | 1.215 (0.008) | 1.227 (0.008) |  | 1.070 (0.008) | 1.047 (0.008) |  |  |
| % change | 0.21% | 0.11% | 0.60 | -0.48% | -0.95% | 0.10 | 0.33 |
| Trabecular Bone Score | | | | | | | |
| Baseline | 1.314 (0.008) | 1.353 (0.007) |  | 1.294 (0.007) | 1.310 (0.007) |  |  |
| 2-year | 1.312 (0.008) | 1.337 (0.007) |  | 1.274 (0.007) | 1.290 (0.007) |  |  |
| % change | -0.20% | -1.15% | 0.045 | -1.51% | -1.52% | 0.94 | 0.17 |

Supplemental Table 2. Baseline Vitamin K Measures, divided by the median, and 2-year Changes in Bone Turnover, adjusted

for age, sex, and randomization group

| **Bone Turnover Marker** | **PK** | | | **%ucOC** | | | **(dp)ucMGP** | | |
| --- | --- | --- | --- | --- | --- | --- | --- | --- | --- |
|  | **<median (0.90 nmol/L; low vitamin K status)** | **≥median**  **(high vitamin K status)** | **p-value** | **≥median**  **(63.8%; low vitamin K status)** | **<median**  **(high vitamin K status)** | **p-value** | **≥median**  **(497.4 pmol/L; low vitamin K status)** | **<median**  **(high vitamin K status)** | **p-value** |
| P1NP, mcg/L | | | | | | | | | |
| Baseline | 47.40 (1.26) | 49.81  (1.18) |  | 45.05 (1.12) | 54.09 (1.27) |  | 47.03 (1.23) | 50.32 (1.22) |  |
| 2-year | 50.64 (1.39) | 50.91  (1.31) |  | 48.04 (1.26) | 54.86 (1.42) |  | 50.58 (1.36) | 51.00 (1.35) |  |
| % change | 6.84% | 2.20% | 0.15 | 6.66% | 1.42% | 0.14 | 7.55% | 1.34% | 0.052 |
| Total OC, ng/mL | | | | | | | | | |
| Baseline | 16.06 (0.35) | 15.26 (0.32) |  | 13.24 (0.32) | 18.09 (0.32) |  | 16.25 (0.34) | 14.96 (0.34) |  |
| 2-year | 17.34 (0.40) | 15.84 (0.36) |  | 15.18 (0.38) | 17.94 (0.38) |  | 17.42 (0.39) | 15.60 (0.38) |  |
| % change | 7.97% | 3.85% | 0.091 | 14.71% | -0.83% | <0.001 | 7.18% | 4.29% | 0.20 |
| CTX, ng/mL | | | | | | | | | |
| Baseline | 0.41 (0.01) | 0.40 (0.01) |  | 0.38 (0.01) | 0.44 (0.01) |  | 0.39 (0.01) | 0.42 (0.01) |  |
| 2-year | 0.41 (0.01) | 0.42 (0.01) |  | 0.38 (0.01) | 0.46 (0.01) |  | 0.41 (0.01) | 0.42 (0.01) |  |
| % change | -0.53% | 5.33% | 0.089 | 2.00% | 3.21% | 0.63 | 3.73% | 1.41% | 0.52 |

Supplemental Table 3. Baseline Vitamin K Measures, divided by clinical significance, and 2-year Changes in Bone Density, Structure, and Turnover, adjusted for age, sex, and randomization group

| **Bone Measure** | **PK** | | | | | **(dp)ucMGP** | | | | |
| --- | --- | --- | --- | --- | --- | --- | --- | --- | --- | --- |
|  | **≤0.5 nmol/L (low vitamin K status)** | | **>0.5 nmol/L**  **(higher vitamin K status)** | | **p-value** | **>300 pmol/L (lower vitamin K status)** | | **≤300 pmol/L (high vitamin K status)** | | **p-value** |
|  | n | Mean (SE) | n | Mean (SE) |  | n | Mean (SE) | n | Mean (SE) |  |
| Trabecular Bone Score | | | | | | | | | | |
| Baseline | 140 | 1.317 (0.008) | 531 | 1.319 (0.004) |  | 649 | 1.317 (0.004) |  | 1.353 (0.019) |  |
| 2-year | 121 | 1.297 (0.008) | 470 | 1.306 (0.004) |  | 570 | 1.303 (0.004) |  | 1.348 (0.020) |  |
| % change |  | -1.51% |  | -0.95% | 0.19 |  | -1.09% |  | -0.32% | 0.42 |
| Volumetric Bone Density and Structure, as assessed by pQCT | | | | | | | | | | |
| Radius | | | | | | | | | | |
| Total vBMD (4%), mg/cm^3^ | | | | | | | | | | |
| Baseline | 151 | 370.35 (5.22) | 496 | 370.58 (2.86) |  | 626 | 371.19 (2.54) | 21 | 350.73 (13.92) |  |
| 2-year | 129 | 374.84 (5.58) | 431 | 377.35 (3.05) |  | 541 | 377. 10 (2.72) | 19 | 367.10 (14.77) |  |
| % change |  | 1.21% |  | 1.83% | 0.50 |  | 1.59% |  | 4.67% | 0.19 |
| Trabecular vBMD (4%), mg/cm^3^ | | | | | | | | | | |
| Baseline | 151 | 202.77 (3.20) | 496 | 197.94 (1.75) |  | 626 | 199.08 (1.56) | 21 | 198.43 (8.55) |  |
| 2-year | 129 | 204.34 (3.43) | 431 | 197.96 (1.88) |  | 541 | 199.54 (1.68) | 19 | 196.18 (9.13) |  |
| % change |  | 0.77% |  | 0.01% | 0.22 |  | 0.23% |  | -1.14% | 0.36 |
| Cortical vBMD (33%), mg/cm^3^ | | | | | | | | | | |
| Baseline | 145 | 1200.4 (2.44) | 474 | 1196.2 (1.34) |  | 600 | 1197.2 (1.19) | 19 | 1195.5 (6.63) |  |
| 2-year | 119 | 1201.4 (2.55) | 396 | 1198.9 (1.40) |  | 469 | 1199.5 (1.24) | 19 | 1198.3 (6.76) |  |
| % change |  | 0.09% |  | 0.23% | 0.29 |  | 0.19% |  | 0.24% | 0.88 |
| Cortical Thickness (33%), mm | | | | | | | | | | |
| Baseline | 145 | 3.26 (0.03) | 474 | 3.24 (0.02) |  | 600 | 3.25 (0.02) | 19 | 3.07 (0.09) |  |
| 2-year | 119 | 3.20 (0.03) | 396 | 3.19 (0.02) |  | 496 | 3.20 (0.02) | 19 | 3.03 (0.09) |  |
| % change |  | -1.60% |  | 01.61% | 0.97 |  | -1.63% |  | -1.11% | 0.42 |
| Tibia | | | | | | | | | | |
| Total vBMD (4%), mg/cm^3^ | | | | | | | | | | |
| Baseline | 154 | 295.58 (3.48) | 507 | 296.65 (1.91) |  | 639 | 296.67 (1.70) | 22 | 288.40 (9.31) |  |
| 2-year | 134 | 295.35 (3.55) | 444 | 297. 30 (1.95) |  | 558 | 297.01 (1.73) | 20 | 291.77 (9.49) |  |
| % change |  | -0.08% |  | 0.22% | 0.20 |  | 0.11% |  | 1.17% | 0.058 |
| Trabecular vBMD (4%), mg/cm^3^ | | | | | | | | | | |
| Baseline | 154 | 248.51 (3.11) | 507 | 247.67 (1.71) |  | 639 | 248.12 (1.52) | 22 | 240.10 (8.32) |  |
| 2-year | 134 | 249.59 (3.18) | 444 | 248.77 (1.75) |  | 558 | 249.22 (1.55) | 20 | 241.28 (8.51) |  |
| % change |  | 0.43% |  | 0.44% | 0.97 |  | 0.44% |  | 0.49% | 0.95 |
| Cortical vBMD (38%), mg/cm^3^ | | | | | | | | | | |
| Baseline | 150 | 1167.5 (2.46) | 501 | 1163.3 (1.35) |  | 629 | 1164.3 (1.20) | 22 | 1165.5 (6.57) |  |
| 2-year | 135 | 1169.5 (2.50) | 444 | 1166.4 (1.37) |  | 559 | 1167.1 (1.22) | 20 | 1168.1 (6.66) |  |
| % change |  | 0.17% |  | 0.26% | 0.27 |  | 0.24% |  | 0.23% | 0.93 |
| Cortical Thickness (38%), mm | | | | | | | | | | |
| Baseline | 150 | 5.59 (0.06) | 501 | 5.64 (0.03) |  | 629 | 5.63 (0.03) | 22 | 5.49 (0.15) |  |
| 2-year | 135 | 5.54 (0.06) | 444 | 5.60 (0.03) |  | 559 | 5.59 (0.03) | 20 | 4.50 (0.16) |  |
| % change |  | -0.88% |  | -0.59% | 0.13 |  | -0.69% |  | 0.12% | 0.061 |
| Bone Strength Indices, as assessed by pQCT | | | | | | | | | | |
| Bone Strength Index, radius (4%), mg*mm | | | | | | | | | | |
| Baseline | 151 | 46.49 (1.05) | 496 | 45.62 (0.58) |  | 626 | 45.91 (0.51) | 21 | 43.39 (2.81) |  |
| 2-year | 129 | 46.75 (1.10) | 431 | 46.21 (0.60) |  | 541 | 46.36 (0.54) | 19 | 45.77 (2.93) |  |
| % change |  | 0.56% |  | 1.30% | 0.44 |  | 0.98% |  | 5.49% | 0.055 |
| Bone Strength Index, tibia (4%), mg*mm | | | | | | | | | | |
| Baseline | 154 | 103.69 (2.25) | 507 | 105.20 (1.24) |  | 639 | 104.98 (1.10) | 22 | 101.06 (6.03) |  |
| 2-year | 134 | 104.03 (2.29) | 444 | 105.62 (1.26) |  | 558 | 105.34 (1.12) | 20 | 102.36 (6.12) |  |
| % change |  | 0.33% |  | 0.39% | 0.87 |  | 0.34% |  | 1.28% | 0.34 |
| Polar Stress Strength Index, radius (33%), mm^3^ | | | | | | | | | | |
| Baseline | 145 | 286.00 (5.04) | 474 | 284.47 (2.76) |  | 600 | 284.14 (2.45) | 19 | 305.52 (13.59) |  |
| 2-year | 119 | 287.25 (5.12) | 396 | 284.88 (2.80) |  | 496 | 284.61 (2.49) | 19 | 309.77 (13.73) |  |
| % change |  | 0.44% |  | 0.14% | 0.48 |  | 0.16% |  | 1.39% | 0.17 |
| Polar Stress Strength Index, tibia (38%), mm^3^ | | | | | | | | | | |
| Baseline | 150 | 1878.6 (28.05) | 501 | 1874.6 (15.38) |  | 629 | 1872.4 (13.69) | 22 | 1968.5 (74.79) |  |
| 2-year | 135 | 1879.7 (27.98) | 444 | 1884.5 (15.35) |  | 559 | 1879.8 (13.65) | 20 | 1989.6 (74.57) |  |
| % change |  | 0.06% |  | 0.53% | 0.059 |  | 0.40% |  | 1.07% | 0.20 |
| Bone Turnover Markers | | | | | | | | | | |
| P1NP, mcg/L | | | | | | | | | | |
| Baseline | 93 | 46.33 (1.78) | 307 | 49.39 (0.98) |  | 384 | 48.14 (0.87) | 16 | 61.52 (4.33) |  |
| 2-year | 89 | 51.86 (1.07) | 293 | 50.45 (1.08) |  | 366 | 50.57 (0.96) | 16 | 56.15 (4.75) |  |
| % change |  | 11.93% |  | 2.15% | 0.011 |  | 5.03% |  | -8.72% | 0.035 |
| Total OC, ng/mL | | | | | | | | | | |
| Baseline | 166 | 16.32 (0.51) | 596 | 15.42 (0.27) |  | 739 | 15.64 (0.24) | 24 | 14.53 (1.36) |  |
| 2-year | 147 | 17.63 (0.58) | 536 | 16.21 (0.31) |  | 661 | 16.57 (0.28) | 22 | 14.67 (1.54) |  |
| % change |  | 8.03% |  | 5.08% | 0.29 |  | 5.90% |  | 0.92% | 0.49 |
| CTX, ng/mL | | | | | | | | | | |
| Baseline | 93 | 0.41 (0.02) | 306 | 0.40 (0.01) |  | 383 | 0.41 (0.01) | 16 | 0.36 (0.04) |  |
| 2-year | 89 | 0.40 (0.02) | 293 | 0.42 (0.01) |  | 366 | 0.41 (0.01) | 16 | 0.42 (0.05) |  |
| % change |  | -0.89% |  | 3.59% | 0.26 |  | 1.97% |  | 17.44% | 0.11 |

Supplemental Table 4. Baseline Vitamin K Measures, first quartile (≤Q1) versus fourth quartile (>Q3), and 2-year Changes in Bone Density and Structure, adjusted for age, sex, and randomization group

| **Bone Measure** | **PK** | | | **%ucOC** | | | **(dp)ucMGP** | | |
| --- | --- | --- | --- | --- | --- | --- | --- | --- | --- |
|  | **≤Q1 (0.60 nmol/L; low vitamin K status)** | **>Q3 (1.50 nmol/L; high vitamin K status)** | **p-value** | **>Q3**  **(78.6%; low vitamin K status)** | **≤Q3**  **(52.6%; high vitamin K status)** | **p-value** | **>Q3**  **(604.8 pmol/L; low vitamin K status)** | **≤Q1**  **(415.5 pmol/L; high vitamin K status)** | **p-value** |
| Areal Bone Mineral Density, g/cm^2^, mean (SE) | | | | | | | | | |
| Spine | | | | | | | | | |
| Baseline | 1.019 (0.011) | 1.020 (0.013) |  | 1.033 (0.012) | 0.982 (0.012) |  | 1.041 (0.013) | 1.007 (0.013) |  |
| 2-year | 1.019 (0.012) | 1.025 (0.014) |  | 1.034 (0.012) | 0.983 (0.012) |  | 1.051 (0.013) | 1.005 (0.013) |  |
| % change | 0.08% | 0.45% | 0.34 | 0.09% | 0.03% | 0.88 | 0.91% | -0.25% | 0.005 |
| Total Hip | | | | | | | | | |
| Baseline | 0.922 (0.008) | 0.931 (0.010) |  | 0.949 (0.009) | 0.888 (0.009) |  | 0.951 (0.009) | 0.909 (0.010) |  |
| 2-year | 0.912 (0.008) | 0.924 (0.010) |  | 0.941 (0.009) | 0.880 (0.009) |  | 0.941 (0.010) | 0.901 (0.010) |  |
| % change | -1.09% | -0.81% | 0.31 | -0.83% | -0.86% | 0.92 | -1.00% | -0.81% | 0.42 |
| Femoral Neck | | | | | | | | | |
| Baseline | 0.758 (0.008) | 0.761 (0.009) |  | 0.779 (0.008) | 0.735 (0.008) |  | 0.778 (0.009) | 0.754 (0.009) |  |
| 2-year | 0.751 (0.008) | 0.756 (0.009) |  | 0.776 (0.009) | 0.729 (0.009) |  | 0.769 (0.009) | 0.749 (0.009) |  |
| % change | -0.84% | -0.66% | 0.65 | -0.45% | -0.80% | 0.44 | -1.12% | -0.55% | 0.15 |
| Whole Body | | | | | | | | | |
| Baseline | 1.143 (0.007) | 1.146 (0.009) |  | 1.148 (0.008) | 1.125 (0.008) |  | 1.146 (0.008) | 1.142 (0.008) |  |
| 2-year | 1.138 (0.007) | 1.143 (0.009) |  | 1.147 (0.008) | 1.122 (0.008) |  | 1.145 (0.008) | 1.138 (0.008) |  |
| % change | -0.43% | -0.26% | 0.48 | -0.11% | -0.28% | 0.43 | -0.13% | -0.30% | 0.49 |
| Trabecular Bone Score | | | | | | | | | |
| Baseline | 1.318 (0.007) | 1.313 (0.008) |  | 1.305 (0.007) | 1.325 (0.007) |  | 1.295 (0.009) | 1.336 (0.008) |  |
| 2-year | 1.299 (0.007) | 1.305 (0.008) |  | 1.291 (0.008) | 1.309 (0.007) |  | 1.286 (0.009) | 1.321 (0.008) |  |
| % change | -1.45% | -0.67% | 0.14 | -1.05% | -1.21% | 0.71 | -0.75% | -1.16% | 0.41 |
| Volumetric Bone Density and Structure | | | | | | | | | |
| Radius | | | | | | | | | |
| Total vBMD (4%), mg/cm^3^ | | | | | | | | | |
| Baseline | 374.50 (4.63) | 376.44 (5.91) |  | 382.46 (4.85) | 358.30 (5.49) |  | 373.48 (5.64) | 369.84 (5.43) |  |
| 2-year | 376.60 (4.86) | 387.92 (6.18) |  | 388.04 (5.19) | 360.90 (5.91) |  | 376.08 (5.88) | 376.54 (5.62) |  |
| % change | 0.56% | 3.05% | 0.017 | 1.46% | 0.73% | 0.49 | 0.69% | 1.81% | 0.31 |
| Trabecular vBMD (4%), mg/cm^3^ | | | | | | | | | |
| Baseline | 201.49 (2.75) | 199.42 (3.50) |  | 202.45 (2.90) | 192.14 (3.29) |  | 202.16 (3.33) | 196.24 (3.21) |  |
| 2-year | 202.67 (2.85) | 200.06 (3.63) |  | 202.35 (3.10) | 194.31 (3.52) |  | 205.51 (3.63) | 195.25 (3.49) |  |
| % change | 0.58% | 0.32% | 0.71 | -0.05% | 1.13% | 0.17 | 1.66% | -0.51% | 0.013 |
| Cortical vBMD (33%), mg/cm^3^ | | | | | | | | | |
| Baseline | 1201.4 (2.14) | 1196.1 (2.75) |  | 1202.2 (2.26) | 1188.6 (2.58) |  | 1196.6 (2.53) | 1196.5 (2.44) |  |
| 2-year | 1203.0 (2.16) | 1199.1 (2.78) |  | 1202.3 (2.37) | 1192.3 (2.71) |  | 1198.2 (2.64) | 1198.3 (2.51) |  |
| % change | 0.14% | 0.25% | 0.42 | 0.01% | 0.31% | 0.069 | 0.13% | 0.15% | 0.91 |
| Cortical Thickness (33%), mm | | | | | | | | | |
| Baseline | 3.28 (0.03) | 3.23 (0.04) |  | 3.30 (0.03) | 3.13 (0.04) |  | 3.27 (0.04) | 3.22 (0.03) |  |
| 2-year | 3.22 (0.03) | 3.19 (0.04) |  | 3.25 (0.03) | 3.06 (0.04) |  | 3.22 (0.04) | 3.16 (0.04) |  |
| % change | -1.81% | -1.24% | 0.11 | -1.49% | -2.19% | 0.16 | -1.54% | -1.79% | 0.58 |
| Tibia | | | | | | | | | |
| Total vBMD (4%), mg/cm^3^ | | | | | | | | | |
| Baseline | 297.12 (3.00) | 297.08 (3.77) |  | 301.42 (3.18) | 288.32 (3.61) |  | 300.10 (3.60) | 292.58 (3.49) |  |
| 2-year | 296.79 (3.03) | 297.41 (3.82) |  | 301.40 (3.23) | 288.02 (3.66) |  | 299.89 (3.69) | 293.58 (3.57) |  |
| % change | -0.11% | 0.11% | 0.46 | -0.01% | -0.10% | 0.74 | -0.07% | 0.34% | 0.20 |
| Trabecular vBMD (4%), mg/cm^3^ | | | | | | | | | |
| Baseline | 249.00 (2.68) | 246.55 (3.38) |  | 249.98 (2.88) | 241.95 (3.27) |  | 252.38 (3.19) | 243.48 (3.09) |  |
| 2-year | 250.03 (2.73) | 247.17 (3.44) |  | 251.25 (2.96) | 242.37 (3.36) |  | 253.76 (3.27) | 244.07 (3.17) |  |
| % change | 0.41% | 0.25% | 0.57 | 0.51% | 0.18% | 0.24 | 0.55% | 0.24% | 0.29 |
| Cortical vBMD (38%), mg/cm^3^ | | | | | | | | | |
| Baseline | 1168.3 (2.00) | 1165.2 (2.51) |  | 1166.5 (2.34) | 1159.0 (2.66) |  | 1162.7 (2.51) | 1164.7 (2.43) |  |
| 2-year | 1170.2 (2.05) | 1167.6 (2.58) |  | 1167.6 (2.40) | 1162.4 (2.73) |  | 1165.7 (2.56) | 1167.8 (2.46) |  |
| % change | 0.16% | 0.20% | 0.67 | 0.10% | 0.29% | 0.056 | 0.26% | 0.27% | 0.96 |
| Cortical Thickness (38%), mm | | | | | | | | | |
| Baseline | 5.60 (0.05) | 5.65 (0.06) |  | 5.61 (0.05) | 5.55 (0.06) |  | 5.58 (0.06) | 5.63 (0.06) |  |
| 2-year | 5.56 (0.05) | 5.62 (0.06) |  | 5.58 (0.06) | 5.50 (0.06) |  | 5.54 (0.06) | 5.59 (0.06) |  |
| % change | -0.77% | -0.64% | 0.59 | -0.52% | -0.88% | 0.10 | -0.70% | -0.72% | 0.92 |
| Bone Strength Indices | | | | | | | | | |
| Bone Strength Index, radius (4%), mg*mm | | | | | | | | | |
| Baseline | 46.69 (0.89) | 45.92 (1.13) |  | 46.80 (0.98) | 43.89 (1.11) |  | 46.31 (1.11) | 46.13 (1.07) |  |
| 2-year | 46.64 (0.92) | 46.98 (1.17) |  | 47.10 (1.03) | 44.24 (1.17) |  | 46.45 (1.16) | 46.69 (1.11) |  |
| % change | -0.11% | 2.30% | 0.025 | 0.63% | 0.79% | 0.92 | 0.31% | 1.22% | 0.40 |
| Bone Strength Index, tibia (4%), mg*mm | | | | | | | | | |
| Baseline | 103.91 (1.91) | 103.58 (2.40) |  | 106.25 (2.06) | 100.77 (2.34) |  | 107.76 (2.27) | 102.04 (2.20) |  |
| 2-year | 104.16 (1.93) | 103.69 (2.43) |  | 106.73 (2.10) | 100.85 (2.39) |  | 107.70 (2.31) | 102.56 (2.24) |  |
| % change | 0.24% | 0.11% | 0.80 | 0.45% | 0.08% | 0.45 | -0.05% | 0.50% | 0.30 |
| Polar Stress Strength Index, radius (33%), mm^3^ | | | | | | | | | |
| Baseline | 283.97 (4.29) | 287.48 (5.50) |  | 277.94 (4.71) | 284.00 (5.39) |  | 288.80 (4.89) | 287.01 (4.70) |  |
| 2-year | 285.11 (4.34) | 287.21 (5.57) |  | 278.35 (4.78) | 284.70 (5.47) |  | 288.40 (5.02) | 288.59 (4.82) |  |
| % change | 0.40% | -0.09% | 0.35 | 0.15% | 0.25% | 0.85 | -0.14% | 0.55% | 0.16 |
| Polar Stress Strength Index, tibia (38%), mm^3^ | | | | | | | | | |
| Baseline | 1862.2 (24.91) | 1877.3 (31.36) |  | 1835.4 (24.78) | 1886.6 (28.15) |  | 1865.7 (27.14) | 1892.6 (26.24) |  |
| 2-year | 1867.4 (24.93) | 1891.8 (31.39) |  | 1839.7 (25.02) | 1898.9 (28.42) |  | 1864.3 (27.17) | 1905.7 (26.25) |  |
| % change | 0.28% | 0.77% | 0.10 | 0.24% | 0.65% | 0.14 | -0.08% | 0.69% | 0.007 |

Supplemental Table 5. Baseline Vitamin K and D Measures, divided at the median, and 2-year Changes in Bone Turnover, adjusted for age, sex, and randomization group

| **Bone Turnover Marker** | **PK and 25(OH)D** | | | | | **%ucOC and 25(OH)D** | | | | | **dp(uc)MGP and 25(OH)D** | | | | |
| --- | --- | --- | --- | --- | --- | --- | --- | --- | --- | --- | --- | --- | --- | --- | --- |
|  | Low vitamin K (PK <0.90 nmol/L) and low vitamin D (25[OH]D <70 nmol/L) status | | High vitamin K (PK ≥0.90 nmol/L) and high vitamin D (25[OH]D ≥70 nmol/L) status | | p-value | Low vitamin K (%ucOC ≥63.8%) and low vitamin D (25[OH]D <70 nmol/L) status | | High vitamin K (%ucOC <63.8%) and high vitamin D (25[OH]D ≥70 nmol/L) status | | p-value | Low vitamin K ([dp]ucMGP ≥497.4 pmol/L) and low vitamin D (25[OH]D <70 nmol/L) status | | High vitamin K ([dp]ucMGP <497.4 pmol/L) and high vitamin D (25[OH]D ≥70 nmol/L) status | | p-value |
|  | n | mean (SE) | n | mean (SE) |  | n | mean (SE) | n | mean (SE) |  | n | mean (SE) | n | mean (SE) |  |
| P1NP, mcg/L | | | | | | | | | | | | | | | |
| Baseline | 99 | 45.17 (1.82) | 113 | 49.45 (1.68) |  | 111 | 43.62 (1.59) | 89 | 53.93 (1.78) |  | 100 | 45.73 (1.78) | 102 | 50.41 (1.78) |  |
| 2-year | 94 | 48.51 (1.98) | 109 | 51.10 (1.82) |  | 104 | 47.04 (1.82) | 84 | 54.94 (2.04) |  | 94 | 49.01 (1.99) | 99 | 51.26 (1.96) |  |
| % change |  | 7.39% |  | 3.34% | 0.39 |  | 7.83% |  | 1.89% | 0.25 |  | 7.16% |  | 1.68% | 0.26 |
| Total OC, ng/mL | | | | | | | | | | | | | | | |
| Baseline | 183 | 15.83 (0.49) | 226 | 14.81 (0.43) |  | 187 | 13.54 (0.46) | 189 | 17.91 (0.46) |  | 195 | 16.12 (0.45) | 200 | 14.50 (0.44) |  |
| 2-year | 160 | 16.85 (0.57) | 204 | 15.56 (0.50) |  | 163 | 15.12 (0.57) | 170 | 17.70 (0.56) |  | 167 | 16.98 (0.51) | 180 | 15.24 (0.50) |  |
| % change |  | 6.42% |  | 5.04% | 0.61 |  | 11.66% |  | -1.17% | 0.001 |  | 5.37% |  | 5.12% | 0.82 |
| CTX, ng/mL | | | | | | | | | | | | | | | |
| Baseline | 99 | 0.40 (0.02) | 113 | 0.41 (0.02) |  | 111 | 0.37 (0.02) | 89 | 0.46 (0.02) |  | 100 | 0.38 (0.02) | 102 | 0.42 (0.02) |  |
| 2-year | 94 | 0.39 (0.01) | 109 | 0.43 (0.02) |  | 104 | 0.38 (0.2) | 84 | 0.48 (0.02) |  | 94 | 0.39 (0.02) | 99 | 0.43 (0.02) |  |
| % change |  | -3.21% |  | 5.50% | 0.034 |  | 1.80% |  | 4.86% | 0.43 |  | 1.69% |  | 2.68% | 0.80 |

Supplemental Table 6. Effect Modification of Supplemental Vitamin D on 2-Year Changes in Trabecular Bone Score (TBS) and Bone Turnover Markers by Baseline Vitamin K Measures, adjusted for age, sex, and randomization group

|  | | | **Vitamin D Group** | | | **Placebo Group** | | | **p, treatment effect** | **p for interaction** |
| --- | --- | --- | --- | --- | --- | --- | --- | --- | --- | --- |
|  |  |  | **N** | **Difference (SE)** | **p-value** | **N** | **Difference (SE)** | **p-value** |  |  |
| **PK** | TBS | Trabecular Bone Score | | | | | | |  | 0.42 |
|  |  | <median (0.90 nmol/L) | 184 | -0.021 (0.005) | <0.001 | 162 | -0.012 (0.005) | 0.018 | 0.20 |  |
|  |  | ≥median | 204 | -0.012 (0.004) | 0.004 | 220 | -0.011 (0.004) | 0.010 | 0.81 |  |
|  | Bone Turnover Markers | P1NP, mcg/L | | | | | | |  | 0.67 |
|  |  | <median (0.90 nmol/L) | 184 | 4.34 (1.56) | 0.006 | 162 | 2.04 (1.62) | 0.21 | 0.31 |  |
|  |  | ≥median | 204 | 1.60 (1.40) | 0.26 | 220 | 0.59 (1.38) | 0.64 | 0.61 |  |
|  |  | Total OC, ng/mL | | | | | | |  | 0.84 |
|  |  | <median (0.90 nmol/L) | 184 | 1.62 (0.51) | 0.001 | 162 | 0.89 (0.55) | 0.11 | 0.33 |  |
|  |  | ≥median | 204 | 0.90 (0.31) | 0.003 | 220 | 0.31 (0.30) | 0.30 | 0.16 |  |
|  |  | CTX, ng/mL | | | | | | |  | 0.44 |
|  |  | <median (0.90 nmol/L) | 184 | 0.005 (0.014) | 0.75 | 162 | -0.010 (0.015) | 0.51 | 0.48 |  |
|  |  | ≥median | 204 | 0.018 (0.013) | 0.18 | 220 | 0.025 (0.013) | 0.06 | 0.71 |  |
| **%ucOC** | TBS | Trabecular Bone Score | | | | | | |  | 0.76 |
|  |  | ≥median (63.8%) | 183 | -0.014 (0.005) | 0.004 | 194 | -0.010 (0.005) | 0.030 | 0.60 |  |
|  |  | <median | 195 | -0.019 (0.004) | <0.001 | 181 | -0.013 (0.005) | 0.005 | 0.29 |  |
|  | Bone Turnover Markers | P1NP, mcg/L | | | | | | |  | 0.090 |
|  |  | ≥median (63.8%) | 183 | 3.07 (1.53) | 0.046 | 194 | 2.93 (1.41) | 0.038 | 0.95 |  |
|  |  | <median | 195 | 3.06 (1.40) | 0.030 | 181 | -2.21 (1.59) | 0.17 | 0.014 |  |
|  |  | Total OC, ng/mL | | | | | | |  | 0.95 |
|  |  | ≥median (63.8%) | 183 | 2.34 (0.36) | <0.001 | 194 | 1.61 (0.34) | <0.001 | 0.14 |  |
|  |  | <median | 195 | 0.23 (0.43) | 0.59 | 181 | -0.56 (0.46) | 0.23 | 0.21 |  |
|  |  | CTX, ng/mL | | | | | | |  | 0.73 |
|  |  | ≥median (63.8%) | 183 | 0.01 (0.01) | 0.31 | 194 | 0.00 (0.01) | 0.84 | 0.54 |  |
|  |  | <median | 195 | 0.02 (0.01) | 0.31 | 181 | 0.01 (0.02) | 0.41 | 0.96 |  |
| **(dp)ucMGP** | TBS | Trabecular Bone Score | | | | | | |  | 0.83 |
|  |  | ≥median (497.4 pmol/L) | 188 | -0.012 (0.005) | 0.013 | 197 | -0.008 (0.005) | 0.087 | 0.58 |  |
|  |  | <median | 200 | -0.020 (0.004) | <0.001 | 186 | -0.014 (0.004) | 0.001 | 0.33 |  |
|  | Bone Turnover Markers | P1NP, mcg/L | | | | | | |  | 0.90 |
|  |  | ≥median (497.4 pmol/L) | 188 | 4.30 (1.53) | 0.006 | 197 | 2.77 (1.54) | 0.074 | 0.48 |  |
|  |  | <median | 200 | 1.61 (1.41) | 0.26 | 186 | -0.29 (1.43) | 0.84 | 0.35 |  |
|  |  | Total OC, ng/mL | | | | | | |  | 0.10 |
|  |  | ≥median (497.4 pmol/L) | 188 | 1.85 (0.50) | <0.001 | 197 | 0.47 (0.49) | 0.35 | 0.048 |  |
|  |  | <median | 200 | 0.69 (0.30) | 0.023 | 186 | 0.63 (0.31) | 0.043 | 0.89 |  |
|  |  | CTX, ng/mL | | | | | | |  | 0.39 |
|  |  | ≥median (497.4 pmol/L) | 188 | 0.02 (0.02) | 0.15 | 197 | 0.01 (0.02) | 0.62 | 0.51 |  |
|  |  | <median | 200 | 0.00 (0.01) | 0.91 | 186 | 0.01 (0.01) | 0.37 | 0.58 |  |

Supplemental Table 7. Effect Modification of Supplemental Vitamin D on 2-Year Changes in Areal Bone Mineral Density, Trabecular Bone Score, and Bone Turnover Markers by Year 2 Vitamin K Measures, adjusted for age, sex, and randomization group

|  | | | **Vitamin D Group** | | | **Placebo Group** | | | **p, treatment effect** | **p for interaction** |
| --- | --- | --- | --- | --- | --- | --- | --- | --- | --- | --- |
|  |  |  | **N** | **Difference (SE)** | **p-value** | **N** | **Difference (SE)** | **p-value** |  |  |
| **PK** | aBMD | Spine, g/cm^2^ | | | | | | |  | 0.78 |
|  |  | <median (0.90 nmol/L) | 176 | 0.002 (0.003) | 0.50 | 156 | 0.001 (0.003) | 0.64 | 0.91 |  |
|  |  | ≥median | 170 | 0.004 (0.003) | 0.12 | 182 | 0.002 (0.003) | 0.39 | 0.61 |  |
|  |  | Total Hip, g/cm^2^ | | | | | | |  | 0.33 |
|  |  | <median (0.90 nmol/L) | 176 | -0.006 (0.002) | <0.001 | 156 | -0.009 (0.002) | <0.001 | 0.22 |  |
|  |  | ≥median | 170 | -0.008 (0.002) | <0.001 | 182 | -0.008 (0.002) | <0.001 | 0.94 |  |
|  |  | Femoral Neck, g/cm^2^ | | | | | | |  | 0.89 |
|  |  | <median (0.90 nmol/L) | 176 | -0.001 (0.002) | 0.75 | 156 | -0.004 (0.002) | 0.089 | 0.31 |  |
|  |  | ≥median | 170 | -0.003 (0.002) | 0.097 | 182 | -0.006 (0.002) | 0.002 | 0.37 |  |
|  |  | Whole Body, g/cm^2^ | | | | | | |  | 0.32 |
|  |  | <median (0.90 nmol/L) | 176 | -0.002 (0.002) | 0.28 | 156 | -0.003 (0.002) | 0.096 | 0.66 |  |
|  |  | ≥median | 170 | -0.003 (0.002) | 0.093 | 182 | -0.001 (0.002) | 0.70 | 0.34 |  |
|  | TBS | Trabecular Bone Score | | | | | | |  | 0.39 |
|  |  | <median (0.90 nmol/L) | 176 | -0.016 (0.005) | <0.001 | 156 | -0.015 (0.005) | 0.004 | 0.83 |  |
|  |  | ≥median | 170 | -0.018 (0.004) | <0.001 | 182 | -0.008 (0.004) | 0.052 | 0.14 |  |
|  | Bone Turnover Markers | P1NP, mcg/L | | | | | | |  | 0.66 |
|  |  | <median (0.90 nmol/L) | 176 | 3.02 (1.67) | 0.072 | 156 | 1.84 (1.67) | 0.27 | 0.62 |  |
|  |  | ≥median | 170 | 2.98 (1.26) | 0.019 | 182 | 0.47 (1.29) | 0.72 | 0.17 |  |
|  |  | Total OC, ng/mL | | | | | | |  | 0.95 |
|  |  | <median (0.90 nmol/L) | 176 | 1.39 (0.48) | 0.004 | 156 | 0.69 (0.51) | 0.18 | 0.32 |  |
|  |  | ≥median | 170 | 1.06 (0.33) | 0.001 | 182 | 0.41 (0.32) | 0.20 | 0.16 |  |
|  |  | CTX, ng/mL | | | | | | |  | 0.23 |
|  |  | <median (0.90 nmol/L) | 176 | 0.011 (0.014) | 0.43 | 156 | 0.025 (0.014) | 0.077 | 0.48 |  |
|  |  | ≥median | 170 | 0.012 (0.013) | 0.37 | 182 | -0.007 (0.014) | 0.61 | 0.32 |  |
| **%ucOC** | aBMD | Spine, g/cm^2^ | | | | | | |  | 0.45 |
|  |  | ≥median (61.2%) | 153 | 0.011 (0.003) | <0.001 | 187 | 0.007 (0.003) | 0.020 | 0.31 |  |
|  |  | <median | 192 | -0.003 (0.003) | 0.18 | 148 | -0.003 (0.003) | 0.26 | 0.98 |  |
|  |  | Total Hip, g/cm^2^ | | | | | | |  | 0.12 |
|  |  | ≥median (61.2%) | 153 | -0.003 (0.002) | 0.15 | 187 | -0.007 (0.002) | <0.001 | 0.044 |  |
|  |  | <median | 192 | -0.011 (0.002) | <0.001 | 148 | -0.010 (0.002) | <0.001 | 0.81 |  |
|  |  | Femoral Neck, g/cm^2^ | | | | | | |  | 0.59 |
|  |  | ≥median (61.2%) | 153 | 0.001 (0.002) | 0.54 | 187 | -0.003 (0.002) | 0.095 | 0.12 |  |
|  |  | <median | 192 | -0.005 (0.002) | 0.024 | 148 | -0.007 (0.002) | 0.003 | 0.44 |  |
|  |  | Whole Body, g/cm^2^ | | | | | | |  | 0.73 |
|  |  | ≥median (61.2%) | 153 | 0.000 (0.002) | 0.95 | 187 | 0.001 (0.002) | 0.55 | 0.73 |  |
|  |  | <median | 192 | -0.005 (0.002) | 0.008 | 148 | -0.005 (0.002) | 0.009 | 0.85 |  |
|  | TBS | Trabecular Bone Score | | | | | | |  | 0.59 |
|  |  | ≥median (61.2%) | 153 | -0.012 (0.005) | 0.014 | 187 | -0.010 (0.004) | 0.032 | 0.70 |  |
|  |  | <median | 192 | -0.021 (0.004) | <0.001 | 148 | -0.013 (0.005) | 0.007 | 0.24 |  |
|  | Bone Turnover Markers | P1NP, mcg/L | | | | | | |  | 0.74 |
|  |  | ≥median (61.2%) | 153 | 0.97 (1.42) | 0.49 | 187 | -0.19 (1.32) | 0.89 | 0.55 |  |
|  |  | <median | 192 | 5.22 (1.53) | <0.001 | 148 | 3.09 (1.71) | 0.072 | 0.35 |  |
|  |  | Total OC, ng/mL | | | | | | |  | 0.95 |
|  |  | ≥median (61.2%) | 153 | 0.17 (0.35) | 0.63 | 187 | -0.32 (0.32) | 0.31 | 0.30 |  |
|  |  | <median | 192 | 2.09 (0.44) | <0.001 | 148 | 1.66 (0.50) | <0.001 | 0.51 |  |
|  |  | CTX, ng/mL | | | | | | |  | 0.87 |
|  |  | ≥median (61.2%) | 153 | 0.00 (0.01) | 0.84 | 187 | 0.00 (0.01) | 0.80 | 0.99 |  |
|  |  | <median | 192 | 0.03 (0.01) | 0.028 | 148 | 0.03 (0.02) | 0.094 | 0.82 |  |
| **(dp)ucMGP** | aBMD | Spine, g/cm^2^ | | | | | | |  | 0.86 |
|  |  | ≥median (513.2 pmol/L) | 172 | 0.007 (0.003) | 0.020 | 170 | 0.005 (0.003) | 0.092 | 0.69 |  |
|  |  | <median | 174 | 0.000 (0.003) | 0.88 | 168 | -0.001 (0.003) | 0.70 | 0.87 |  |
|  |  | Total Hip, g/cm^2^ | | | | | | |  | 0.74 |
|  |  | ≥median (513.2 pmol/L) | 172 | -0.007 (0.002) | <0.001 | 170 | -0.009 (0.002) | <0.001 | 0.45 |  |
|  |  | <median | 174 | -0.007 (0.002) | <0.001 | 168 | -0.008 (0.002) | <0.001 | 0.69 |  |
|  |  | Femoral Neck, g/cm^2^ | | | | | | |  | 0.55 |
|  |  | ≥median (513.2 pmol/L) | 172 | -0.002 (0.002) | 0.44 | 170 | -0.006 (0.002) | 0.007 | 0.17 |  |
|  |  | <median | 174 | -0.002 (0.002) | 0.24 | 168 | -0.004 (0.002) | 0.045 | 0.55 |  |
|  |  | Whole Body, g/cm^2^ | | | | | | |  | 0.40 |
|  |  | ≥median (513.2 pmol/L) | 172 | -0.002 (0.002) | 0.22 | 170 | 0.000 (0.002) | 0.93 | 0.42 |  |
|  |  | <median | 174 | -0.003 (0.002) | 0.13 | 168 | -0.004 (0.002) | 0.047 | 0.73 |  |
|  | TBS | Trabecular Bone Score | | | | | | |  | 0.41 |
|  |  | ≥median (513.2 pmol/L) | 172 | -0.017 (0.005) | <0.001 | 170 | -0.015 (0.005) | 0.002 | 0.82 |  |
|  |  | <median | 174 | -0.017 (0.004) | <0.001 | 168 | -0.008 (0.004) | 0.084 | 0.13 |  |
|  | Bone Turnover Markers | P1NP, mcg/L | | | | | | |  | 0.77 |
|  |  | ≥median (513.2 pmol/L) | 172 | 3.46 (1.71) | 0.044 | 170 | 2.08 (1.72) | 0.23 | 0.57 |  |
|  |  | <median | 174 | 2.54 (1.23) | 0.039 | 168 | 0.27 (1.25) | 0.83 | 0.20 |  |
|  |  | Total OC, ng/mL | | | | | | |  | 0.087 |
|  |  | ≥median (513.2 pmol/L) | 172 | 2.01 (0.50) | <0.001 | 170 | 0.61 (0.51) | 0.22 | 0.050 |  |
|  |  | <median | 174 | 0.46 (0.29) | 0.11 | 168 | 0.45 (0.29) | 0.12 | 0.99 |  |
|  |  | CTX, ng/mL | | | | | | |  | 0.35 |
|  |  | ≥median (513.2 pmol/L) | 172 | 0.01 (0.02) | 0.59 | 170 | 0.02 (0.02) | 0.23 | 0.63 |  |
|  |  | <median | 174 | 0.01 (0.01) | 0.20 | 168 | 0.00 (0.01) | 0.97 | 0.36 |  |
